# Diagnostic performance of combined biomarkers and phonocardiography vs. the 2024 ESC risk factor-weighted clinical likelihood model for detecting coronary artery disease

**DOI:** 10.1093/ehjimp/qyag043

**Published:** 2026-03-10

**Authors:** Marie Muthspiel, Mahdi Mahmoudi, Moritz Hebein, Christoph C Kaufmann, Achim Leo Burger, Amro Ahmed, Ben Panzer, Paul F Harbich, Philipp Hohensinner, Johann Wojta, Kurt Huber, Alexander Geppert, Bernhard Jäger

**Affiliations:** 3rd Medical Department with Cardiology and Intensive Care Medicine, Clinic Ottakring, Montleartstraße 37, 1160 Vienna, Austria; Faculty of Medicine, Sigmund Freud University, Freudplatz 1, 1020 Vienna, Austria; Faculty of Medicine, Sigmund Freud University, Freudplatz 1, 1020 Vienna, Austria; Ludwig Boltzmann Institute for Lung Health, Sanatoriumstraße 2, 1140 Vienna, Austria; 3rd Medical Department with Cardiology and Intensive Care Medicine, Clinic Ottakring, Montleartstraße 37, 1160 Vienna, Austria; 3rd Medical Department with Cardiology and Intensive Care Medicine, Clinic Ottakring, Montleartstraße 37, 1160 Vienna, Austria; Faculty of Medicine, Sigmund Freud University, Freudplatz 1, 1020 Vienna, Austria; 3rd Medical Department with Cardiology and Intensive Care Medicine, Clinic Ottakring, Montleartstraße 37, 1160 Vienna, Austria; Faculty of Medicine, Sigmund Freud University, Freudplatz 1, 1020 Vienna, Austria; 3rd Medical Department with Cardiology and Intensive Care Medicine, Clinic Ottakring, Montleartstraße 37, 1160 Vienna, Austria; 3rd Medical Department with Cardiology and Intensive Care Medicine, Clinic Ottakring, Montleartstraße 37, 1160 Vienna, Austria; 3rd Medical Department with Cardiology and Intensive Care Medicine, Clinic Ottakring, Montleartstraße 37, 1160 Vienna, Austria; Department of Internal Medicine II, Medical University of Vienna, Spitalgasse 23, Vienna 1090, Austria; Ludwig Boltzmann Institute for Cardiovascular Research, Schwarzspanierstraße 17, 1090 Vienna, Austria; Department of Internal Medicine II, Medical University of Vienna, Spitalgasse 23, Vienna 1090, Austria; Ludwig Boltzmann Institute for Cardiovascular Research, Schwarzspanierstraße 17, 1090 Vienna, Austria; 3rd Medical Department with Cardiology and Intensive Care Medicine, Clinic Ottakring, Montleartstraße 37, 1160 Vienna, Austria; Faculty of Medicine, Sigmund Freud University, Freudplatz 1, 1020 Vienna, Austria; Ludwig Boltzmann Institute for Cardiovascular Research, Schwarzspanierstraße 17, 1090 Vienna, Austria; 3rd Medical Department with Cardiology and Intensive Care Medicine, Clinic Ottakring, Montleartstraße 37, 1160 Vienna, Austria; 3rd Medical Department with Cardiology and Intensive Care Medicine, Clinic Ottakring, Montleartstraße 37, 1160 Vienna, Austria; Faculty of Medicine, Sigmund Freud University, Freudplatz 1, 1020 Vienna, Austria; Department of Internal Medicine II, Medical University of Vienna, Spitalgasse 23, Vienna 1090, Austria; Ludwig Boltzmann Institute for Cardiovascular Research, Schwarzspanierstraße 17, 1090 Vienna, Austria

**Keywords:** coronary artery disease, pre-test probability, non-invasive testing, CADScor©System, phonocardiography, risk factor-weighted clinical likelihood model

## Abstract

**Aims:**

Non-invasive rule-out of coronary artery disease (CAD) using phonocardiography (CADScor©System) has been introduced as an alternative to the calculation of clinical risk-scores in populations with low-to intermediate CAD-likelihood. This study aims to (i) evaluate the diagnostic performance of the CADScor©System in a population with an intermediate CAD-likelihood and (ii) investigate whether a combined strategy of selected biomarkers and use of the device improves diagnostic accuracy compared to the 2024 ESC risk factor-weighted clinical likelihood (RF-CL) model.

**Methods and results:**

A total of 167 patients with symptoms suggesting CAD and scheduled for coronary computed tomography angiography (CCTA) or elective invasive coronary angiography (ICA) were prospectively enrolled. Blood samples and heart sound recordings, obtained with the CADScor©System, were performed within 24 h before scheduled examinations. A resulting score (CADScore) ranging from 0 to 99 determined the risk of CAD. Diagnostic performance was calculated as sensitivity, specificity, negative predictive value, and positive predictive value. Diagnostic accuracy incorporating cardiovascular biomarkers was assessed using AUC-ROC analysis. Bootstrap validation (*n* = 2000 resamples) was performed to assess estimate stability. Net Reclassification Index (NRI) and Decision Curve Analysis (DCA) were conducted to evaluate reclassification performance and clinical utility. Obstructive CAD was detected in 56 (33.6%) patients. The diagnostic performance of the CADScor©System at a cut-off value of ≤20 was characterized by a negative predictive value of 90.0% (95% confidence interval [CI], 74.2–96.6), a positive predictive value of 37.8% (95% CI, 34.6–41.1), a sensitivity of 93.8% (95% CI, 82.8–98.7), and a specificity of 26.7% (95% CI, 18.4–36.5), respectively. Diagnostic accuracy of the CADScor©System was defined by an AUC of 0.743 (95% CI, 0.656–0.830), while the RF-CL model demonstrated an AUC of 0.719 (95% CI, 0.629–0.807, *P* = 0.559). Bootstrap validation confirmed estimate stability, with no statistically significant differences between methods. Net Reclassification Index analysis showed modest improvement with CADScore (Total NRI = 0.039), and decision curve analysis demonstrated clinical utility for both models. High-sensitivity troponin I (hs-TnI) was a significant predictor of obstructive CAD. Combining hs-TnI values with CADScore using logistic regression yielded an AUC of 0.768 (95% BCa CI, 0.670–0.842), showing no superior diagnostic performance compared to the RF-CL model.

**Conclusion:**

The CADScor©System is a reliable non-invasive test method for ruling out obstructive CAD in a population with intermediate disease prevalence, characterized by high sensitivity (93.8%) and negative predictive value (90.0%). Bootstrap validation confirmed the stability of diagnostic performance estimates across all methods. While incorporation of hs-TnI as a predictor for obstructive CAD did not significantly improve diagnostic accuracy beyond the CADScore alone, Net Reclassification Index analysis revealed modest improvements in patient classification. Decision curve analysis demonstrated complementary clinical utility patterns between both models. The 2024 ESC RF-CL model remains a strong primary approach for risk stratification, while CADScore offers excellent rule-out capability.

**Trial registration number:**

EK 21-012-0221

## Graphical Abstract

**Graphical Abstract:**
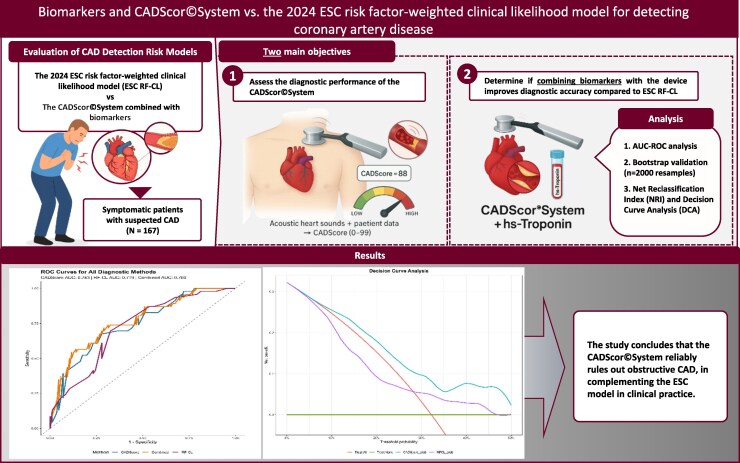

## Introduction

Coronary artery disease (CAD) is one of the leading causes of death, accounting for 16% of deaths worldwide.^1^ Therefore, risk stratification and diagnostic workup of patients with suspected obstructive CAD is of particular importance. However, currently available non-invasive and invasive test methods are characterized by high financial costs, associated risks, and low diagnostic yield.^2–5^ Thus, radiation-free diagnostic tools with low complication rates providing high negative predictive value are needed to rapidly rule out obstructive CAD. Different approaches, such as the risk factor-weighted clinical likelihood (RF-CL) model presented in the 2024 ESC guidelines for the management of chronic coronary syndromes (CCS), attempt to improve the clinical likelihood of CAD, which in previous models was usually associated with an overestimation of the actual disease prevalence.^6,7^ Another method is offered by the CADScor©System (Acarix A/S), a diagnostic tool based on the principle of phonocardiography.^8^ Schmidt *et al*. calculated a negative predictive value (NPV) of 97.2% and a positive predictive value (PPV) of 13.7% in a population with low- to intermediate CAD likelihood.^9^ Compared to the updated Diamond-Forester Score (2019 ESC Guidelines), the CADScor©System reclassified 472 of 1395 low- to intermediate-risk patients as <15% risk while increasing significant CAD risk in the reclassified group from 3.1% to 4.0%.^9^ These results indicated a significant improvement compared to the recommended standard diagnostics of previous guidelines. However, studies to date have predominantly enrolled patients with low- to intermediate likelihood of CAD and the diagnostic performance of the CADScor©System in comparison with the recently published 2024 ESC risk factor-weighted clinical likelihood (RF-CL) model has not yet been investigated. Additionally, certain cardiovascular biomarkers play a crucial role in predicting CAD. High-sensitivity troponin (hs-Tn) has emerged as an independent predictor for obstructive CAD and appears to complement the diagnostic workup of patients with suspected coronary stenosis undergoing non-invasive testing.^10^ Linden *et al*. described an association between indices of platelet activation and the prevalence of CAD, noting that patients with coronary stenosis had shortened platelet function analyser (PFA)-100 collagen-epinephrine closure time compared to patients without CAD.^11^ Furthermore, plasma fibrinogen is known to be strongly associated with the risk of CAD, irrespective of its severity.^12,13^ Recognizing the significance of cardiovascular biomarkers in predicting CAD risk, the aims of this study were to (i) evaluate the diagnostic performance of the CADScor©System in a population with intermediate CAD-likelihood and (ii) investigate whether a combined strategy of selected biomarkers and use of the CADScor©System leads to an improvement in diagnostic accuracy compared to currently recommended standard diagnostics.

## Methods

### Study design and population

Patients with symptoms of chest pain or dyspnoea suggestive of CAD who underwent coronary computed tomography angiography (CCTA) or elective invasive coronary angiography (ICA) at the 3rd medical department for Cardiology and Intensive Care Medicine at Clinic Ottakring were prospectively enrolled between September 2021 and June 2023. Exclusion criteria were: (i) age below 30 years, (ii) previously diagnosed CAD, (iii) previous coronary revascularization, coronary artery bypass graft or coronary stenting, (iv) implanted electronic equipment in the area of the heart, (v) non-sinus-rhythm, (vi) contrast agent allergy prohibiting planned testing, (vii) pregnancy, (viii) hs-TnI serum values above the assay-specific cut-off (one value above the 99th percentile of the upper reference limit) indicating acute coronary syndrome (ACS) or myocardial injury due to other mechanisms, (ix) ACS including unstable angina, non-ST-elevation myocardial infarction and ST-elevation myocardial infarction, (x) intake of vasodilators within 2 h leading up to the CAD-score measurement, (xi) anatomical or scar induced hindrance at the fourth left intercostal space, (xii) skin conditions not allowing the application of the CADScor©-patch, (xiii) known allergy for polyacrylate, (xiv) inability to hold breath for about 8 s. All patients signed written informed consent. The study was approved by the authorized local ethics committee (EK 21-012-0221). Study enrollment occurred within 24 h prior to CCTA and ICA, respectively. After initial assessment for baseline data, blood samples were obtained. Subsequently, testing was performed with the CADScor©System. Estimation of clinical likelihood of CAD was calculated by using the 2024 ESC RF-CL model, based on the patient's symptoms, age, and gender, as well as the presence of specific risk factors such as family history, smoking, dyslipidaemia, hypertension, and diabetes. Family history of CAD was defined as 1 or more first-degree relatives with early signs of CAD (men <55 and women <65 years of age). Smoking was defined as a current or past smoker. Dyslipidaemia was identified by use of lipid-lowering medication or an LDL-cholesterol level >116 mg/dL. Hypertension and diabetes were characterized by the use of antihypertensive or diabetes medication or a prior diagnosis at the time of study enrolment.

### CADScor©System

The CADScor©System is a non-invasive, radiation-free medical device. Detailed functioning has been previously described.^8^ Briefly, the device is placed on the patients’ fourth, left intercostal space and records heart murmurs resulting from diastolic post-stenotic turbulent flow over a period of 3 min. Based on age, sex, and hypertension (systolic blood pressure ≥140 mmHg or antihypertensive medication), as well as eight acoustic characteristics, a score from 0 to 99 is calculated, with a score ≤20 as the standard cut-off for low CAD risk (rule-out) and a score >20 for increased CAD risk (further testing).

### Coronary computed tomography angiography

CCTA scans and coronary artery calcium score (CACS) calculation were conducted using a Siemens SOMATOM 128 Zeilen Multislice-CT-Scanner, through a peripheral venous catheter injection of contrast media (approximately 90 mL) was performed. An image-quality check was performed according to local standard operating procedures (SOPs). Stenosis of >50% in at least one coronary artery was considered as haemodynamically impairing (obstructive CAD). If a CCTA scan showed luminal stenosis of ≤50%, obstructive CAD was ruled out. If obstructive CAD was suspected on CCTA, ICA with subsequent revascularization, if indicated, was performed within 4–12 weeks after CCTA. In the absence of obstructive CAD on CCTA, no further testing was performed.

### Invasive coronary angiography

ICA was performed by trained interventional cardiologists at the catheterization laboratory according to local SOPs and contemporary guidelines. Rates of approximately 100–200 mL contrast media were used to visualize coronary flow. Coronary obstruction of ≥90% was considered as haemodynamically impairing. PCI was performed according to the standard of care. For stenoses between 30% and 90%, the indication for percutaneous coronary intervention (PCI) was either determined by fractional flow reserve (FFR) with a cut-off value of ≤0.8 being considered obstructive CAD, or left to the operator's discretion by visual assessment or quantitative coronary angiography (QCA). FFR was performed according to the standard of care. Stenosis <30% were interpreted as non-obstructive CAD. No visual stenoses were classified as normal coronaries arteries.

### Statistical analysis

Normality was assessed using both visual inspection of histograms (see Supplementary data online, *Figures S4*–*S5*) and formal statistical testing with the Shapiro–Wilk test. Continuous normally distributed data were presented as arithmetic mean and standard deviation (SD); not normally distributed data were reported as median and interquartile range (IQR). Categorical data were shown as frequencies (percentages). Baseline characteristics were presented descriptively without statistical comparisons to avoid multiple testing issues and power-related interpretation problems. Sensitivity, specificity, negative predictive value, and positive predictive value were calculated for the CADScor©System. Comparisons between the RF-CL model and the CADScore, including selected biomarkers, were made by calculating the area under the curve receiver operating characteristics (AUC-ROC) using DeLong's test for comparing correlated ROC curves. DeLong's test is a non-parametric method specifically designed for comparing the areas under two or more correlated ROC curves, accounting for the paired nature of the diagnostic data from the same patients (20). Logistic regression (2-tailed) was used to analyse the relationship between CAD diagnosis and baseline characteristics separately. A value of *P* < 0.05 was considered significant and performance values were presented with 95% confidence intervals.

Power analysis was performed to assess our ability to detect clinically meaningful differences between diagnostic methods using reasonable assumptions about effect sizes. Using our sample size of 132 patients (46 cases, 86 controls) and DeLong's test for AUC comparisons, our study had limited power to detect small differences (7–11% power for 0.02–0.03 AUC differences) but moderate power for larger effects (68% power for 0.10 AUC difference).

Bootstrap validation was performed using 2000 bootstrap resamples to assess the stability and reliability of AUC estimates and diagnostic performance metrics. Bootstrap resampling was performed using the bias-corrected and accelerated (BCa) method for confidence interval calculation. Comprehensive visualizations of bootstrap distributions and confidence intervals were generated to demonstrate validation robustness. Net Reclassification Index (NRI) was calculated to quantify the improvement in risk classification when comparing CADScore to the RF-CL model. NRI analysis evaluates the proportion of patients who are appropriately reclassified (upward for cases, downward for controls) minus those inappropriately reclassified. Risk categories were defined as low risk (≤20 for CADScore; very low risk ≤5% for RF-CL) vs. higher risk (>20 for CADScore; >5% for RF-CL). Comprehensive reclassification tables and visualizations were generated to illustrate patient movement between risk categories.

Decision curve analysis was performed to evaluate the clinical utility of CADScore vs. the 2024 ESC RF-CL model across different probability thresholds. The analysis was conducted using the dcurves package in R across threshold probabilities from 0% to 50%, which encompasses clinically relevant decision-making scenarios for coronary artery disease diagnosis.

All statistical analyses were performed with IBM SPSS Statistics Version 27, 29 and R Statistics (R 4.2.1) using pROC and ROCR packages.^14,15^

## Results

### Baseline and imaging characteristics

A total of 170 patients provided informed consent and were enrolled in this study. Three patients had to be excluded due to screening failures, resulting in a study population of 167 patients for final analysis. The mean age was 61.8 ± 11.5 years, and 40.1% (67) were female. Sixty-six (39.5%) patients presented with typical angina, 34 (20.4%) with atypical angina, 35 (21.0%) with non-anginal chest pain, and 32 (19.2%) with dyspnoea as the main symptom leading to further testing. Baseline characteristics are presented in *Table 1*. Sixty-eight (40.7%) patients received CCTA, and 116 (69.5%) patients underwent ICA, with a total of 17 (10.2%) patients having both examinations. Among CCTA patients, the median coronary artery calcium score was 10.1 (IQR: 0–150.1), with significant differences between patients with obstructive CAD [119.2 (96.9–364.7)] vs. those without [0 (0–88.7)]. Obstructive CAD was detected in 56 of 167 (33.6%) patients (*Table 2*).

**Table 1:** Baseline characteristics presented descriptively as number and frequency (*n*, %), mean ± SD or median (IQR), respectively

Characteristic	Total study population *n* = 167	Obstructive CAD *n* = 56	Non-obstructive or no CAD *n* = 111
Age (years)	61.8 ± 11.5	65.8 ± 11.5	59.8 ± 11.1
Females, *n* (%)	67 (40.1%)	14 (25%)	53 (47.7%)
Weight (kg)	80 (20.3)	80 (21)	80 (20)
Height (m)	1.7 ± 0.1	1.7 ± 0.1	1.7 ± 0.1
Body mass index (kg/m^2^)	27.1 (6.7)	26.8 (5)	27.3 (7)
Systolic blood pressure (mmHg)	138.5 (22.5)	145.0 (28)	133.0 (5)
Diastolic blood pressure (mmHg)	83.3 ± 10.4	83.5 ± 8.8	83.3 ± 11.3
Heart rate (bpm)	68.5 ± 11.2	70.2 ± 12.7	67.6 ± 10.2
Arterial hypertension, *n* (%)	122 (73.1%)	44 (78.6%)	78 (70.3%)
Family history of early atherosclerotic CVD^a^, *n* (%)	51 (30.5%)	13 (23.2%)	38 (34.2%)
Coronary artery calcium score^b^, median (IQR)	10.1 (0–150.1)	119.2 (96.9–364.7)	0 (0–88.7)
Main symptom at referral, *n* (%):			
Typical angina	66 (39.5%)	25 (44.6%)	41 (36.9%)
Atypical angina	34 (20.4%)	11 (19.6%)	23 (20.7%)
Non-anginal chest pain	35 (21.0%)	9 (16.1%)	26 (23.4%)
Dyspnoea	32 (19.2%)	11 (19.6%)	21 (18.9%)
Pretest probability (RF-CL model), *n* (%):			
≤5% (very low risk)	25 (15.0%)	2 (3.6%)	23 (20.7%)
>5–15% (low risk)	73 (43.7%)	22 (39.3%)	51 (45.9%)
>15–50% (moderate risk)	68 (40.7%)	32 (57.1%)	36 (32.4%)
Active smoker, *n* (%)	43 (25.7%)	12 (27.9%)	31 (72.1%)
Former smoker, *n* (%)	51 (30.5%)	20 (39.2%)	31 (60.8%)
Never smoker, *n* (%)	72 (43.1%)	23 (41.1%)	49 (44.1%)
Diabetes mellitus, *n* (%)	30 (18.0%)	13 (43.3%)	17 (56.7%)
Lipid-lowering drug, *n* (%)	107 (64.1%)	45 (42.1%)	62 (57.9%)
Platelets (G/L)	241 (43)	237 (49)	253.5(89)
Mean platelet volume (fL)	10.6 (0.5)	10.6 (1)	12.10 (2.6)
Platelet distribution width (fL)	12.3 (2.3)	12.4 (2.1)	12.1 (2.6)
Immature platelet fraction (%)	4.95 (3.7)	5.2 (3.5)	4.80 (3.9)
Prothrombin time (%)	96.0 (21.0)	91.0 (23.0)	98.0 (20.0)
Activated partial thromboplastin time (s)	26.6 ± 9.2	26.7 ± 10.6	26.6 ± 8.3
Fibrinogen (mg/dL)	3.4 (1.1)	3.4(1.0)	3.3 (1.1)
Hs-Troponin-I (µg/L)	7.7 (7.6)	9.6 (11.3)	7.0 (5.1)
Lactate dehydrogenase (U/L)	171 (47.5)	161.0 (40)	178 (44)
Triglycerides (mg/dL)	119 (80.5)	121 (69)	116.50 (91)
LDL-cholesterol (mg/dL)	76.7 (64.5)	72.7 (56.4)	77.3 (63.8)
Total cholesterol (mg/dL)	158.5 (64.5)	152(65)	161.5 (71)
Creatinine (mg/dL)	0.9 (0.3)	1 (0.3)	0.9 (0.2)

CAD, coronary artery disease.

^a^Defined as early CAD in first-degree relatives (men <55, women <65 years of age).

^b^Calcium score available only for CCTA patients (*n* = 68).

**Table 2:** Imaging characteristics (*n*, % or median (IQR))

Characteristic	Value
Coronary CT angiography (***n*** = 68)	
Stenosis ≥50%	23 (33.8)
Coronary artery calcium score	10.1 (0–150.1)
Invasive coronary angiography (***n*** = 116)	
Diameter stenosis <30%	44 (37.9)
Diameter stenosis 30–90%	48 (41.4)
Diameter stenosis >90%	24 (20.7)
Fractional flow reserve (***n*** = 11)	
>0.8	10 (90.9)
≤0.8	1 (9.1)
CAD prevalence (***n*** = 167)	
Obstructive CAD	56 (33.6)
Non-obstructive CAD	46 (27.5)
No CAD	65 (38.9)

CT, computed tomography; CAD, coronary artery disease.

### RF-CL model results

The likelihood of CAD was calculated using the RF-CL model as proposed in the 2024 ESC Guidelines.^6^ Accordingly, a total of 23 (13.8%) patients were classified as very low risk, 75 (44.9%) as low risk, and 69 (41.3%) as moderate risk (*Table 1*).

### CADScor©System: diagnostic performance

A CADScore was calculated in 149 (89.2%) patients (*Table 3*). Due to recording errors, a CADScore could not be obtained in 18 (10.8%) patients. Recording errors were either patient-related (irregular heartbeat [*n* = 2], internal noise too high [*n* = 1]), device-related (inconsistent data analysis [*n* = 10], ambient microphone error [*n* = 2]), operator-related (heartbeat signals too low [*n* = 2]), or due to external circumstances (ambient noise too high [*n* = 1]). No adverse events related to the CADScor©System occurred during this study. The median CADScore was 35(20). Patients suffering from obstructive CAD had a significantly higher CADScore when compared to patients without or with non-obstructive CAD (43(17) vs. 34(22), *P* < 0.001). A CADScore ≤20 was detected in 30 patients (20.1%), while a CADScore >20 was recorded in 119 (79.9%) patients. The RF-CL model categorized 23 patients as very low risk, among whom two (8.7%) exhibited obstructive CAD, while the CADScor©System advised to rule out 30 patients without further testing, of whom three (10%) had obstructive CAD. The diagnostic performance of the CADScor©System at a cut-off value of ≤20 was characterized by a negative predictive value of 90.0% (95% CI,74.2–96.6%), a positive predictive value of 37.8% (95% CI, 34.6–41.1%), a sensitivity of 93.8% (95% CI, 82.8–98.7%) and a specificity of 26.7% (95% CI, 18.4–36.5%) (*Table 3*). Negative and positive predictive values, as well as sensitivity and specificity for various other cut-off values in the present cohort, are shown in *Table 4*.

**Table 3:** Diagnostic performance of the CadScor©System with a cut-off ≤20

CADScore (*n* = 149)	35 (20)	
Obstructive CAD (*n* = 48)	43 (17)	
Non-obstructive or no CAD (*n* = 101)	34 (22)	*P* < 0.001
True negative (*n*)	27	
False negative (*n*)	3	
True positive (*n*)	45	
False positive (*n*)	74	
Negative predictive value	90.0% (74.2–96.6%)	
Positive predictive value	37.8% (34.6–41.1%)	
Sensitivity	93.8% (82.8–98.7%)	
Specificity	26.7% (18.4–36.5%)	

CAD, coronary artery disease.

**Table 4:** Diagnostic performance of the CadScor©System for different cut-off values

Cut-off value	Total patients (*n*)	True negative (*n*)	False negative (*n*)	Sensitivity (%)	Specificity (%)	Positive predictive value (%)	Negative predictive value (%)
14	20	20	0	100% (92.6–100)	19.8% (12.5–28.9)	37.2% (35.0–39.5)	100% (83.2–100)
15	22	21	1	97.9% (88.9–100)	20.8% (13.4–30.0)	37.0% (34.5–39.6)	95.5% (74.4–99.3)
16	25	23	2	95.8% (85.8–99.5)	22.8% (15.0–32.2)	37.1% (34.3–40.0)	92.0% (73.9–97.9)
17	26	24	2	95.8% (85.8–99.5)	23.8% (15.9–33.3)	37.4% (34.6–40.3)	92.3% (74.7–98.0)
18	27	25	2	95.8% (85.8–99.5)	24.8% (16.7–34.3)	37.7% (34.8–40.7)	92.6% (75.5–98.1)
19	28	26	2	95.8% (85.8–99.5)	25.7% (17.6–35.4)	38.0% (35.0–41.1)	92.9% (76.3–98.1)
**20**	**30**	**27**	**3**	**93.8% (82.8–98.7)**	**26.7% (18.4–36.5)**	**37.8% (34.6–41.1)**	**90.0% (74.2–96.6)**
21	31	27	4	91.7% (80.0–97.7)	26.7% (18.4–36.5)	37.3% (34.0–40.8)	87.1% (71.5–94.8)
22	33	29	4	91.7% (80.0–97.7)	28.7% (20.2–38.6)	37.9% (34.5–41.5)	87.9% (73.0–95.1)
23	35	30	5	89.6% (77.3–96.5)	29.7% (21.0–39.6)	37.7% (34.1–41.5)	85.7% (71.3–93.6)
24	35	30	5	89.6% (77.3–96.5)	29.7% (21.0–39.6)	37.7% (34.1–41.5)	85.7% (71.3–93.6)
25	42	35	7	85.4% (72.2- 93.9)	34.7% (25.5–44.8)	38.3% (34.1–42.8)	83.3% (70.6–91.3)
26	43	36	7	85.4% (72.2–93.9)	35.6% (26.4–45.8)	38.7% (34.4–43.2)	83.7% (71.2–91.5)
27	49	40	9	81.3% (67.4–91.1)	39.6% (30.0–49.8)	39.0% (34.2–44.1)	81.6% (70.2–89.4)
28	50	41	9	81.3% (67.4–91.1)	40.6% (30.9–50.8)	39.4% (34.5–44.5)	82.0% (70.7–89.6)
29	53	44	9	81.3% (67.4–91.1)	43.6% (33.7–53.8)	40.6% (35.5–46.0)	83.0% (72.3–90.2)
30	54	45	9	81.3% (67.4–91.1)	44.6% (34.7–54.8)	41.1% (35.8–46.5)	83.3% (72.7–90.4)
31	56	47	9	81.3% (67.4–91.1)	46.5% (36.6–56.7)	41.9% (36.5–47.5)	83.9% (73.7–90.7)

### Incorporation of cardiovascular biomarkers and comparison with the RF-CL model

**Figure 1:**
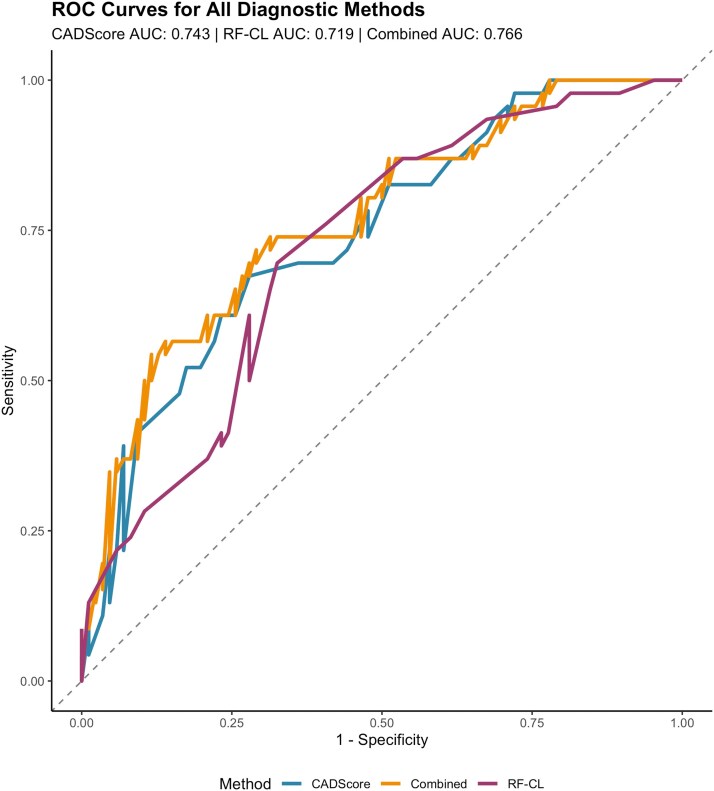
ROC curves comparing diagnostic performance of CADScore, RF-CL model, and combined approach (CADScore + hs-TnI). AUC values with 95% confidence intervals are shown. *P*-values calculated using DeLong's test for comparing correlated ROC curves. AUC, area under the curve; RF-CL, risk factor-weighted clinical likelihood; hs-TnI, high-sensitivity troponin-I.

To correctly compare AUC-ROC analyses, 17 patients were excluded due to missing values, resulting in a final number of 132 patients for AUC calculations. The AUC for the diagnostic performance of the CADScor©System was 0.743 (95% CI, 0.656–0.830) while the RF-CL model revealed an AUC of 0.719 (95% CI, 0.629–0.807, *P* = 0.559). When combining the CADScore with serum hs-TnI, a significant predictor for obstructive CAD in our cohort (*Table 5*), the resulting AUC was 0.766 (95% CI, 0.681–0.851), indicating no significant improvement in diagnostic accuracy compared to the RF-CL model (*P* = 0.324) (*Figure 1*).

**Table 5:** Binary logistic regression of selected risk factors and obstructive coronary artery disease

	*β* (regression coefficient)	Standard error	Odds ratio *(ExpB)*	95% CI for OR	*P*-value
Weight	0.00	0.01	1.00	0.98–1.02	0.76
Height	−0.16	1.76	0.85	0.03–26.83	0.93
Body mass index	−0.01	0.04	1.00	0.93–1.06	0.86
Family history of CVD	0.52	0.38	1.68	0.81–3.51	0.17
Smoke status	−0.19	0.42	0.65	0.36–1.89	0.65
Diabetes mellitus II	−0.51	0.41	0.60	0.27–1.34	0.21
Lipid-lowering drug	1.31	0.41	3.71	1.65–8.35	**0.002**
Platelets	0.00	0.00	1.00	0.99–1.01	0.83
Mean platelet volume	0.01	0.19	1.01	0.71–1.46	0.95
Platelet distribution width	−0.03	0.08	0.97	0.83–1.14	0.72
Immature platelet fraction	0.01	0.05	1.01	0.91–1.13	0.83
Prothrombin time	−0.01	0.01	0.99	0.97–1.01	0.21
Activated partial thromboplastin time	0.00	0.02	1.00	0.97–1.04	0.93
Fibrinogen	0.29	0.23	1.34	0.85–2.11	0.21
Hs-Troponin-I	0.03	0.02	1.03	1.01–1.06	**0.02**
Lactate dehydrogenase	0.00	0.01	1.00	0.99–1.01	0.48
Triglycerides	0.00	0.00	1.00	0.99–1.01	0.63
LDL-cholesterol	−0.01	0.00	0.99	0.99–1.00	0.16
Total cholesterol	−0.01	0.00	0.99	0.99–1.00	0.07

Bold *P*-values are used to show significance. CVD, cardiovascular disease; HS, high-sensitive; LDL, low-density lipoprotein.

**Figure 2:**
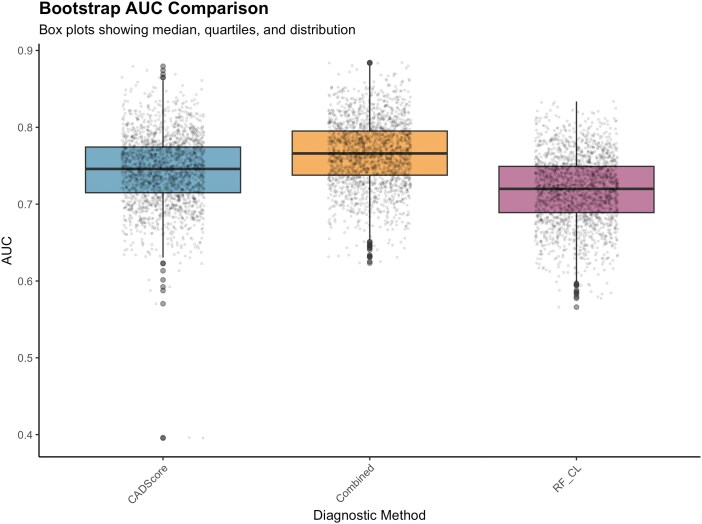
Bootstrap validation of AUC estimates showing box plots from 2000 bootstrap resamples for CADScore, RF-CL model, and combined approach. Box plots demonstrate the stability of AUC estimates with overlapping confidence intervals. Median values and interquartile ranges confirm robust diagnostic performance across bootstrap samples.

**Figure 3:**
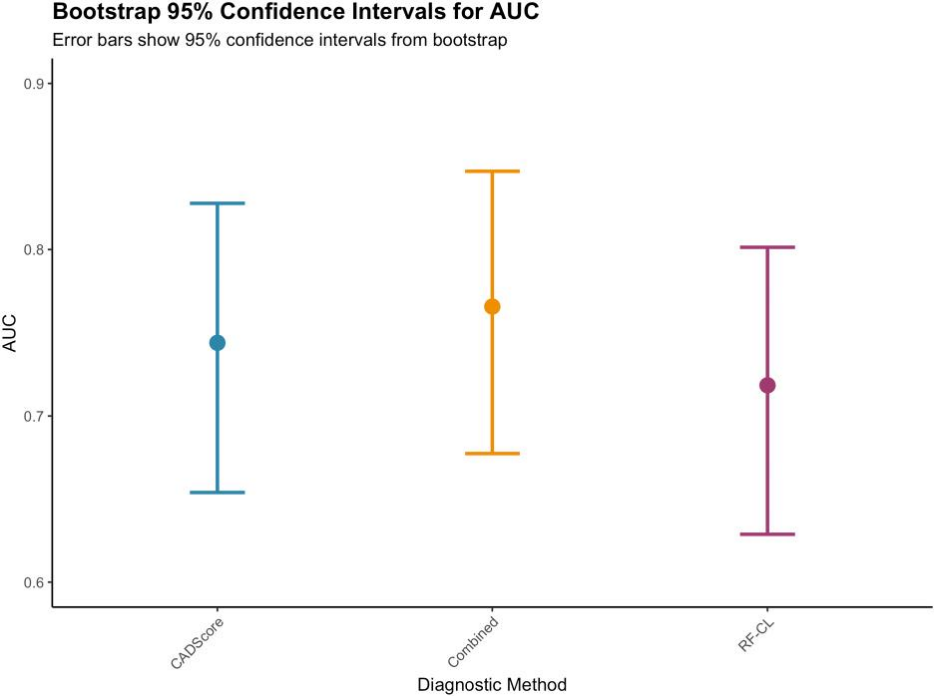
Bootstrap confidence intervals for AUC comparisons. Error bars show 95% confidence intervals for pairwise AUC differences, with all intervals including zero, confirming non-significant differences between diagnostic methods. Bootstrap validation supports statistical conclusions from DeLong's test.

Bootstrap validation (*n* = 2000 resamples) confirmed estimate stability: CADScore AUC 0.744 (95% BCa CI: 0.646–0.821), RF-CL model AUC 0.718 (95% BCa CI: 0.622–0.796), and combined approach AUC 0.768 (95% BCa CI: 0.67–0.842) (*Figure 2*). All pairwise comparisons showed non-significant differences with confidence intervals including zero (*Figure 3*). Additional bootstrap validation results are provided as Supplementary data online, *Figures S6*–*S9*.

**Figure 4:**
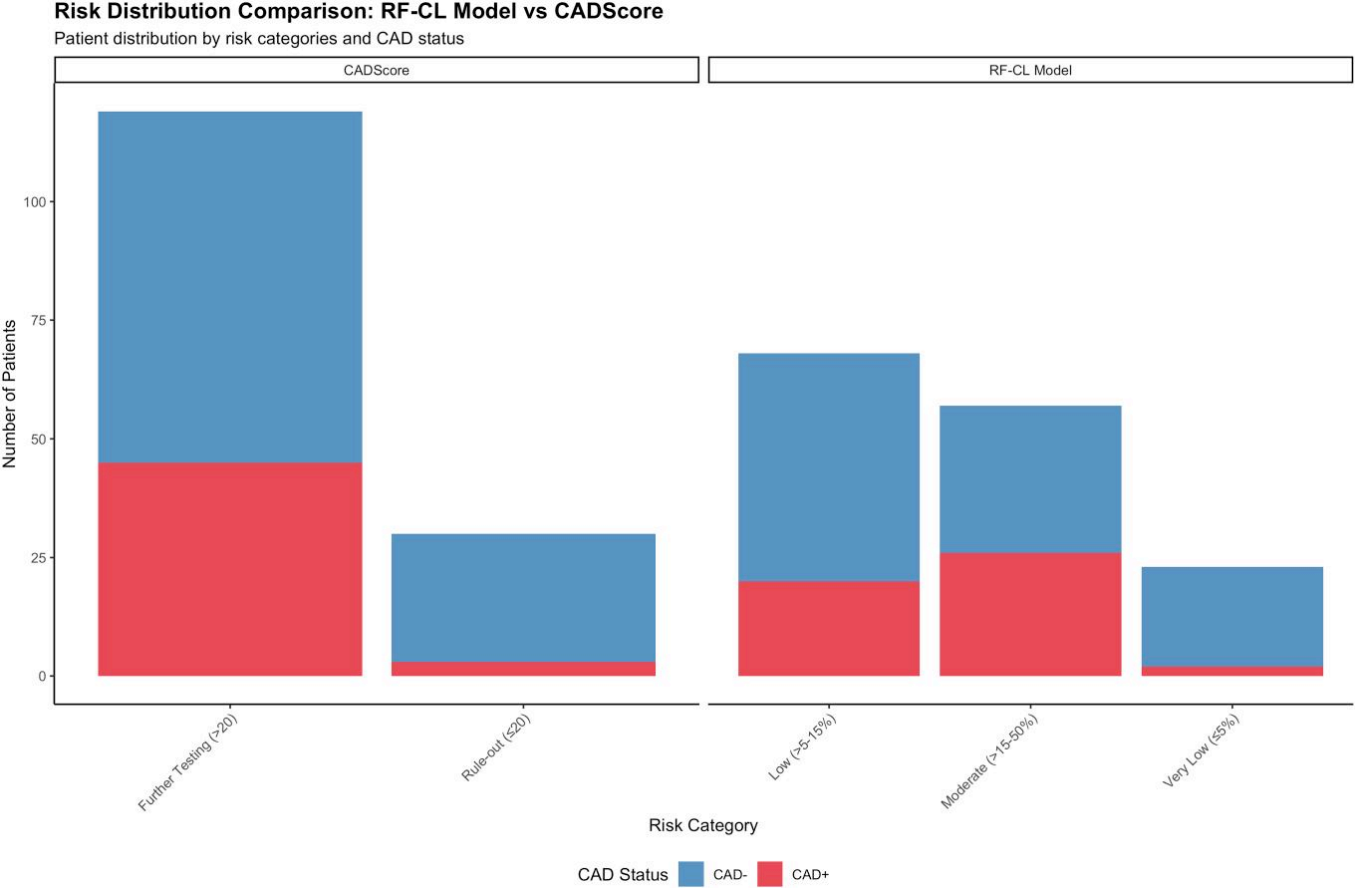
Net reclassification index risk distribution comparison between CADScore and RF-CL model. Side-by-side comparison shows patient distribution across risk categories stratified by CAD status (*n* = 149). CADScore uses binary classification (≤20 rule-out vs. >20 further testing) while the RF-CL model uses three-category classification (≤5% very low, >5–15% low, >15–50% moderate risk). The visualization demonstrates risk redistribution patterns that contribute to the modest NRI improvement (0.039).

**Figure 5:**
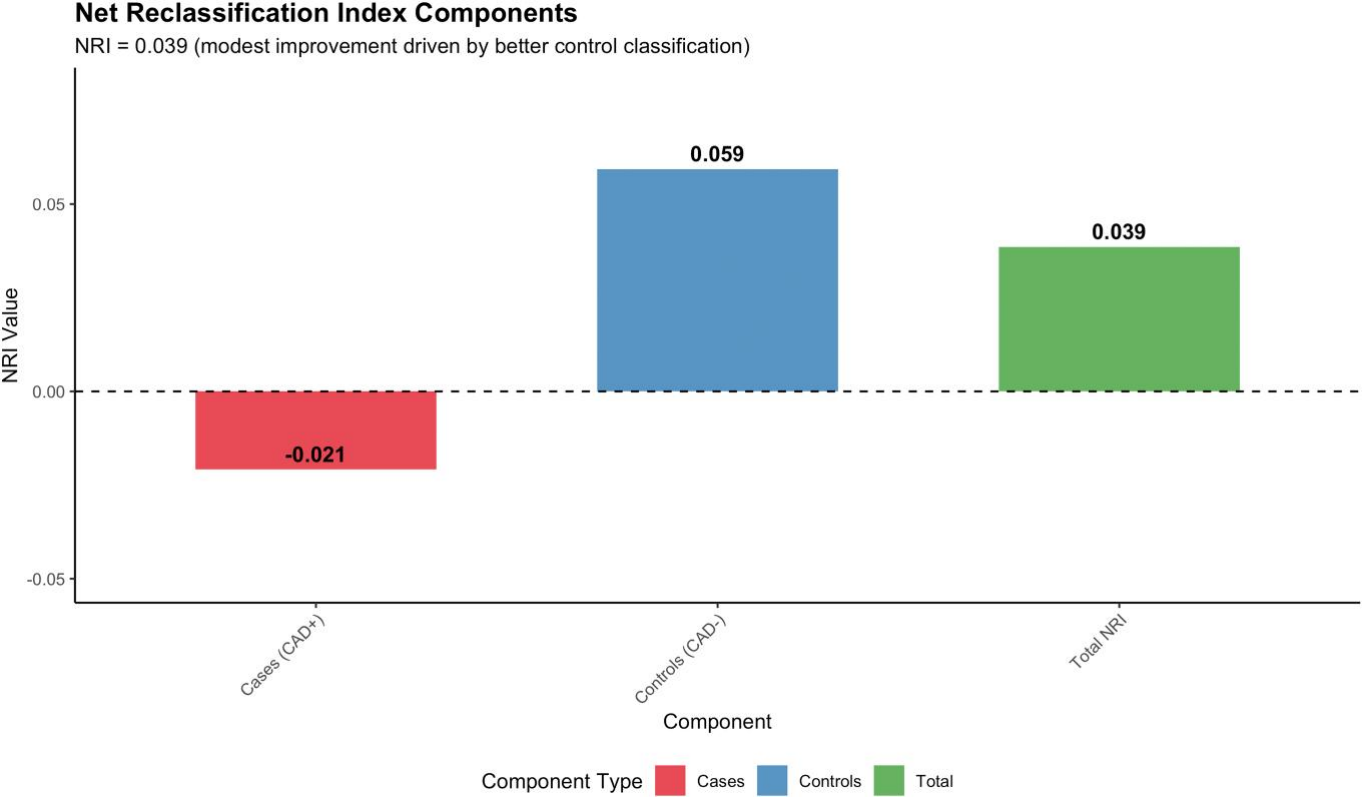
Net reclassification index components breakdown showing individual contributions from cases with obstructive CAD (NRI = −0.021) and controls without obstructive CAD (NRI = +0.059). The bar chart illustrates how the overall NRI improvement (0.039) is primarily driven by better classification of patients without obstructive CAD, while cases show a slight decrease in classification performance. Error bars represent 95% confidence intervals.

Net Reclassification Index (NRI) analysis revealed a modest improvement when using CADScore compared to the RF-CL model (total NRI = 0.039). This improvement was primarily driven by better classification of patients without obstructive CAD (NRI for controls = +0.059), while cases with obstructive CAD showed a slight decrease in classification performance (NRI for cases = −0.021). Detailed reclassification analysis showed that 13 controls were appropriately reclassified to lower risk categories, while seven were inappropriately moved to higher risk, and two cases were appropriately moved to higher risk, while three were inappropriately moved to lower risk. Risk reclassification patterns between the two models are illustrated in *Figures 4–5*, with comprehensive reclassification analysis provided in Supplementary data online, *Figures S1*–*S3*.

**Figure 6:**
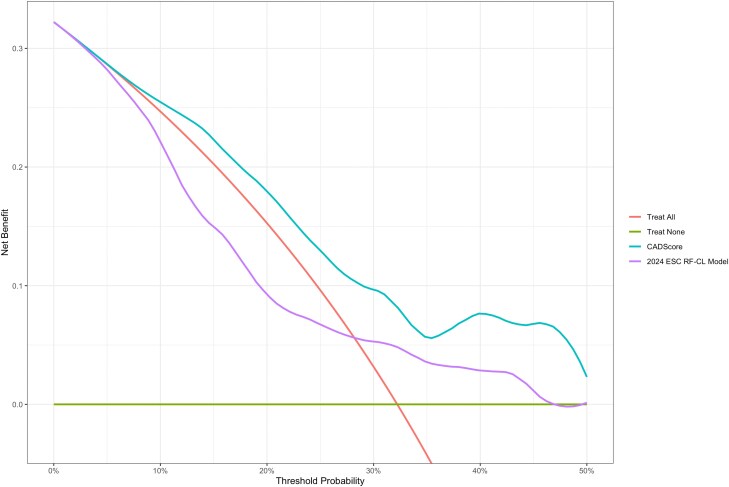
Decision curve analysis comparing CADScore and 2024 ESC risk factor-weighted clinical likelihood (RF-CL) models. The curves show net benefit across probability thresholds from 0% to 50%, demonstrating clinical utility for both models above treat-all and treat-none strategies within clinically relevant threshold ranges. Both models show complementary performance characteristics across different risk thresholds.

Decision curve analysis was performed to evaluate the clinical utility of both models across probability thresholds from zero% to 50% using 149 patients with complete data (48 cases, 101 controls). Both the CADScore and RF-CL model demonstrated clinical utility above the treat-all and treat-none strategies within clinically relevant threshold ranges. The decision curves revealed complementary performance characteristics, with CADScore showing optimal net benefit at specific threshold ranges, particularly effective for intermediate-risk patients, while the RF-CL model demonstrated varying optimal performance across different risk thresholds (*Figure 6*).

## Discussion

The present study is the first to compare the diagnostic performance of the CADScor©System to the predictive RF-CL model, recommended in the recently published 2024 ESC guidelines, in order to estimate the likelihood of obstructive CAD in an intermediate prevalence cohort. We further investigated, for the first time, whether a combined strategy of selected biomarkers and use of the CADScor©System leads to an improvement in diagnostic accuracy.

The theoretical foundation for phonocardiography in CAD detection rests on well-established pathophysiological principles. Coronary stenosis creates haemodynamic disturbances that generate characteristic acoustic signatures through turbulent blood flow patterns, particularly during diastole when post-stenotic flow turbulence is most prominent.^8^ Unlike traditional auscultation, the CADScor©System employs advanced signal processing to detect subtle murmurs inaudible to conventional examination, providing direct functional assessment of coronary stenosis rather than statistical probability estimation based on population risk factors.

With a high sensitivity of 93.8%, the CADScor©System was able to identify 45 of 48 patients with obstructive CAD and therefore performed equally well as in populations with low- to intermediate likelihood.^8,9,16^ The negative predictive value of 90.0% was, as expected, lower than in previously investigated cohorts due to the higher prevalence of CAD in our patients.^8,9,16,17^ The specificity of 26.7% reflects the test's design as a highly sensitive screening tool optimized for rule-out rather than rule-in applications. While this limits the test's ability to avoid unnecessary further testing, the high sensitivity (93.8%) and NPV (90.0%) support its clinical utility for excluding obstructive CAD in symptomatic patients. This is further supported by a robust negative likelihood ratio (NLR) of 0.23 and limited positive likelihood ratio (PLR) of 1.28 (see Supplementary data online, *Table S2*). These metrics underscore the consistent usefulness of the CADScor©System as a rule-out tool and provide evidence of its reliability in populations with varying disease prevalences. Schmidt *et al*.'s landmark study demonstrated the clinical potential of this approach, achieving a remarkable NPV of 97.2% in low-intermediate risk populations and successfully reclassifying 472 of 1395 intermediate-risk patients to lower risk categories, suggesting that acoustic analysis could provide complementary diagnostic information beyond traditional risk stratification. It should further be noted that this study included a higher proportion of patients with atypical-, non-anginal chest pain and dyspnoea (60.6% in total) when compared to previous data. This underscores the efficacy of the CADScor©System not only as a useful rule-out device in patients with typical angina, but also in those with atypical symptoms. In this study, a cut-off ≤20 was chosen to identify patients at increased risk for CAD. However, the present results further indicate that the choice of a lower cut-off value could be particularly beneficial in patients with intermediate CAD prevalence. A lower cut-off value (consequently higher negative predictive value) would reduce the likelihood of false negatives, providing an even safer use of the device in this population.

The RF-CL model has been introduced in the 2024 ESC guidelines for the management of CCS as a new risk stratification tool to estimate the likelihood of obstructive CAD. Former models, like the updated Diamond-Forrester Score (2019 pre-test probability [PTP] model) have been associated with an overestimation of the actual disease prevalence, limiting their use in clinical practice.^6^ Besides gender, age, and angina symptoms, the RF-CL model incorporates additional risk factors (family history, smoking, dyslipidaemia, hypertension, and diabetes), which lead to a threefold increase in the number of patients classified as having very low (≤5%) likelihood of obstructive CAD when compared to the 2019 PTP model.^7^ In the present study, the diagnostic accuracy of the CADScor©System, as characterized by the AUC, did not differ significantly from that of the ESC 2024 RF-CL model. While this suggests comparable diagnostic performance, it also indicates that the device offers no substantial additional prognostic value over the RF-CL model.

Given the non-superiority demonstrated in this study, the CADScor©System's role in contemporary clinical pathways appears limited to specific settings rather than standard cardiology referral centres. Recent data showed that incorporating the CADScor©System into routine diagnostic assessment allowed more patients with very low pre-test probability (≤5%) to safely forgo further testing. However, when applied in outpatient cardiology clinics, it did not lead to a reduction in overall testing, indicating limited impact in specialized centres with established diagnostic pathways.^16^ The device may have potential utility in (i) imaging-limited environments where advanced cardiac imaging is unavailable or delayed, (ii) primary care settings for rapid triage of patients with chest pain symptoms, and (iii) resource-constrained healthcare settings where point-of-care testing could facilitate initial risk stratification. However, in standard cardiology practice with access to guideline-recommended risk stratification tools, the RF-CL model remains the preferred approach due to its simplicity, guideline endorsement, comparable performance, and integration into established clinical workflows. The current evidence does not support routine adoption of CADScor©System in guideline-driven pathways where the RF-CL model is readily available and equally effective.

The scientific rationale for combining biomarkers with phonocardiography stems from their complementary mechanistic pathways: phonocardiography measures haemodynamic consequences of stenosis, while biomarkers reflect biochemical aspects of coronary pathophysiology. High-sensitivity troponin I detects subclinical myocardial injury from demand-supply mismatch, providing molecular evidence of ischaemia that could theoretically enhance acoustic detection of stenosis.^17,18^ Serum TnI was a significant predictor for obstructive CAD in our cohort, consistent with previous data.^18,19^ However, incorporating hs-TnI into the CADScore did not enhance diagnostic accuracy, which remained comparable to the RF-CL model. This neutral result suggests that the acoustic information provided by the device may already capture the functional significance of coronary stenosis, with biomarker addition providing redundant rather than complementary information.

Decision curve analysis provided insights into the clinical utility of both diagnostic approaches beyond traditional performance metrics. Both CADScore and the RF-CL model demonstrated clinical utility above treat-all and treat-none strategies within clinically relevant threshold ranges, with complementary performance characteristics across different probability thresholds. However, it is important to emphasize that complementary net benefit does not equate to superiority, and threshold-dependent utility does not necessarily justify adoption in guideline-driven pathways. The RF-CL model remains the preferred approach in standard cardiology practice given its simplicity, guideline endorsement, and comparable diagnostic performance.

The neutral results observed in our study should be interpreted considering our sample size limitations and the strong performance of both methods. Power analysis indicated limited ability to detect small differences (7–11% power for 0.02–0.03 AUC differences) but adequate power for large differences (68% for >0.10 AUC), suggesting no major differences exist. Bootstrap validation confirmed the stability of our estimates, and the modest NRI improvement (0.039) primarily benefited patients without obstructive CAD. These findings indicate comparable diagnostic performance between methods rather than equivalence, consistent with patterns in diagnostic accuracy research where small differences between well-performing methods require larger samples for statistical significance.

## Strengths and limitations

This study is the first to investigate the diagnostic performance of the CADScor©System in comparison to the RF-CL model. Estimation of likelihood and calculation of the CadScore were performed before assessing actual CAD severity through ICA or CCTA, ensuring a partially blinded approach. However, several important limitations warrant acknowledgment.

### Statistical and sample size limitations

The relatively small sample size (*n* = 167, with 132 patients for AUC analyses) limited our ability to detect modest differences between well-performing diagnostic methods. Power analysis demonstrated limited power to detect small but potentially clinically meaningful AUC differences (7–11% power for 0.02–0.03 differences), indicating that larger studies are needed to definitively establish the comparative performance of these diagnostic approaches. The limited sample size also resulted in wide confidence intervals and reduced precision for comparing well-performing diagnostic methods.

### Study design and generalizability

The single-centre design may limit external validity and generalizability to other healthcare settings, patient populations, or clinical practices. Our intermediate prevalence cohort may not reflect populations with different risk distributions, potentially limiting applicability to broader clinical settings. Further, the study was limited to a cross-sectional design, and follow-up data, such as incidence and timing of revascularization, were not collected. Our findings, therefore, only apply to the models investigated and do not address the impact on therapeutic decisions or clinical outcomes.

### Population and selection considerations

Patients were pre-selected for cardiac imaging (CCTA or ICA), which may have created a referral bias towards higher-risk individuals. The symptom heterogeneity (60.6% with atypical chest pain or dyspnoea) may have affected diagnostic performance for both methods. Missing data necessitated the exclusion of 17 patients from AUC analyses, potentially introducing selection bias.

### Methodological constraints

We compared existing diagnostic tools rather than developing new models, which limited our ability to optimize diagnostic algorithms specifically for our population. The study was not designed according to specific diagnostic accuracy guidelines (e.g. STARD), which may affect interpretability and comparability with other studies.

### Imaging and procedural limitations

The timing between CADScore measurement and definitive cardiac imaging (within 24 h) may have introduced temporal variability in clinical status. Per-vessel territory analysis was not feasible due to study design limitations and insufficient power for subgroup analyses. Future research should evaluate CADScore performance by coronary territory to identify potential vessel-specific diagnostic patterns.

### Clinical applicability

Limited data on reasons for ICA referral and systematic collection of additional clinical variables (e.g. detailed family history, medication effects) may have affected comprehensive risk assessment. The study did not evaluate clinical outcomes or cost-effectiveness, limiting assessment of real-world clinical utility.

## Conclusions

The present study demonstrates that the CADScor©System is a reliable rule-out device in an intermediate prevalence cohort for obstructive CAD, characterized by a high sensitivity of 93.8% and a negative predictive value of 90.0%, falling within the predicted range. However, neither the CADScore alone nor the incorporation of hs-tnI levels in the CADScore algorithm provided a significant benefit in diagnostic accuracy compared to the 2024 ESC RF-CL model. Based on the simplicity of performance, the RF-CL model should present the preferred strategy for prediction of significant CAD in those patients.

Implications for Practice

- **Standard cardiology practice:** The 2024 ESC RF-CL model should be considered the preferred first-line risk stratification tool. It is guideline-endorsed, established in clinical workflows, requires no additional equipment, and demonstrates comparable diagnostic performance to the CADScor©System.
- **CADScor©System potential applications:** The device may be useful in specific settings where (i) advanced cardiac imaging is unavailable or significantly delayed, (ii) rapid point-of-care triage is needed in primary care or emergency departments, or (iii) resource constraints limit access to guideline-recommended risk stratification tools. However, the device does not provide superior diagnostic accuracy compared to the RF-CL model.
- **Combined biomarker approach**: The addition of high-sensitivity troponin I to the CADScore does not improve diagnostic accuracy and is not recommended.

## Supplementary Material

qyag043_Supplementary_Data

## Data Availability

The data underlying this article will be shared on reasonable request to the corresponding author.

## Lead author biography

Dr. Marie Muthspiel completed her medical doctorate in 2021 and is currently working as a cardiology resident at Ottakring Clinic in Vienna. In addition, she is pursuing her PhD at the Medical University of Vienna. Her clinical and research interests are primarily in the field of electrophysiology and preventive cardiovascular medicine.
