# Supplementary material for: Diagnostic performance of combined biomarkers and phonocardiography vs. the 2024 ESC risk factor-weighted clinical likelihood model for detecting coronary artery disease
Source: Eur Heart J Imaging Methods Pract. 2026 Mar 10;4(1):qyag043. doi: 10.1093/ehjimp/qyag043 (PMC13032869; doi:10.1093/ehjimp/qyag043)
Supplement: qyag043_Supplementary_Data [file qyag043_supplementary_data.zip › Supplementary_Figures_total.docx]

SUPPLEMENTARY FIGURES


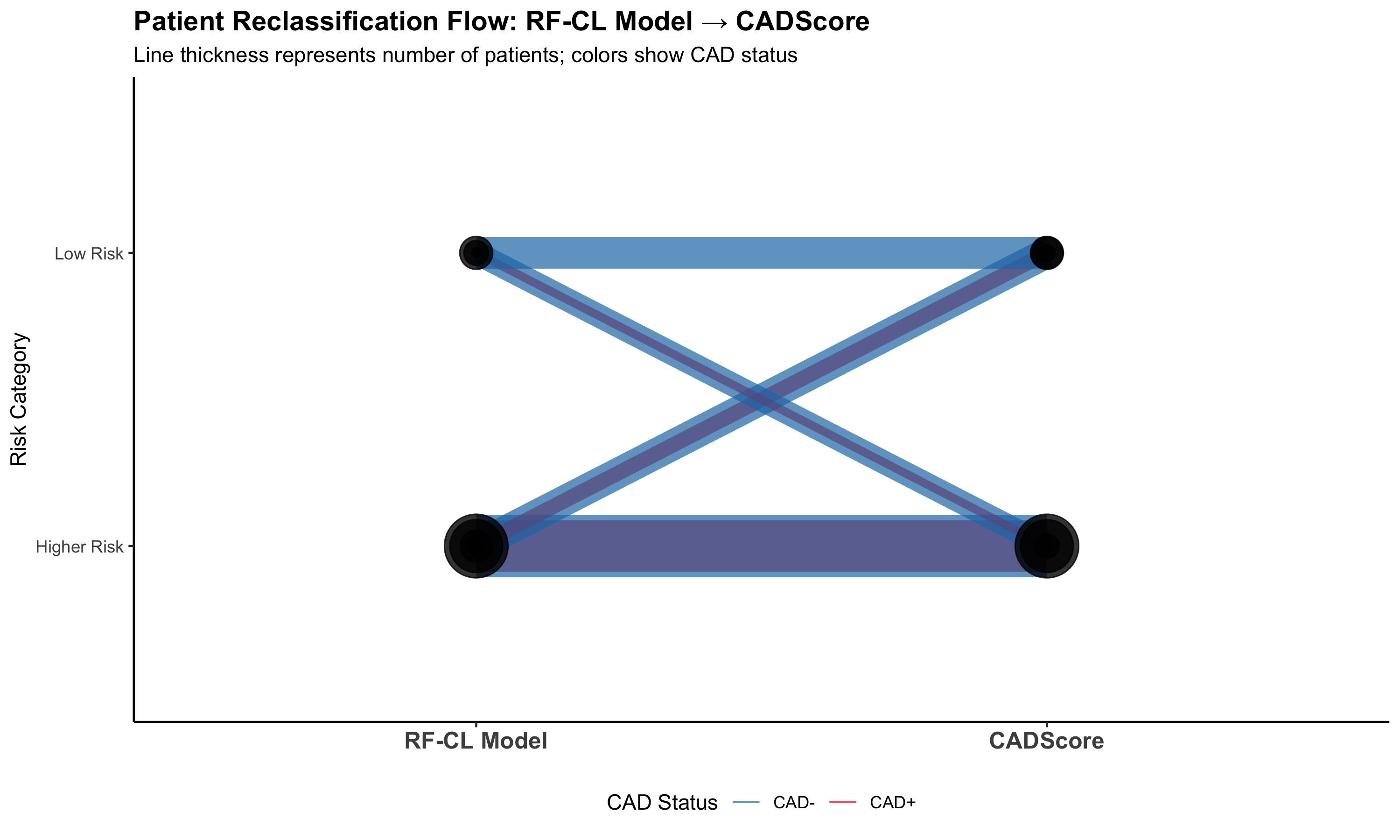


**Supplementary Figure 1:** Net Reclassification Index flow diagram illustrating patient movement between risk categories when transitioning from RF-CL model to CADScore classification. Line thickness represents the number of patients moving between categories, with colors indicating appropriate (green) or inappropriate (red) reclassification. The diagram provides detailed visualization of the 149 patients included in NRI analysis, showing exact patient numbers for each reclassification pathway.


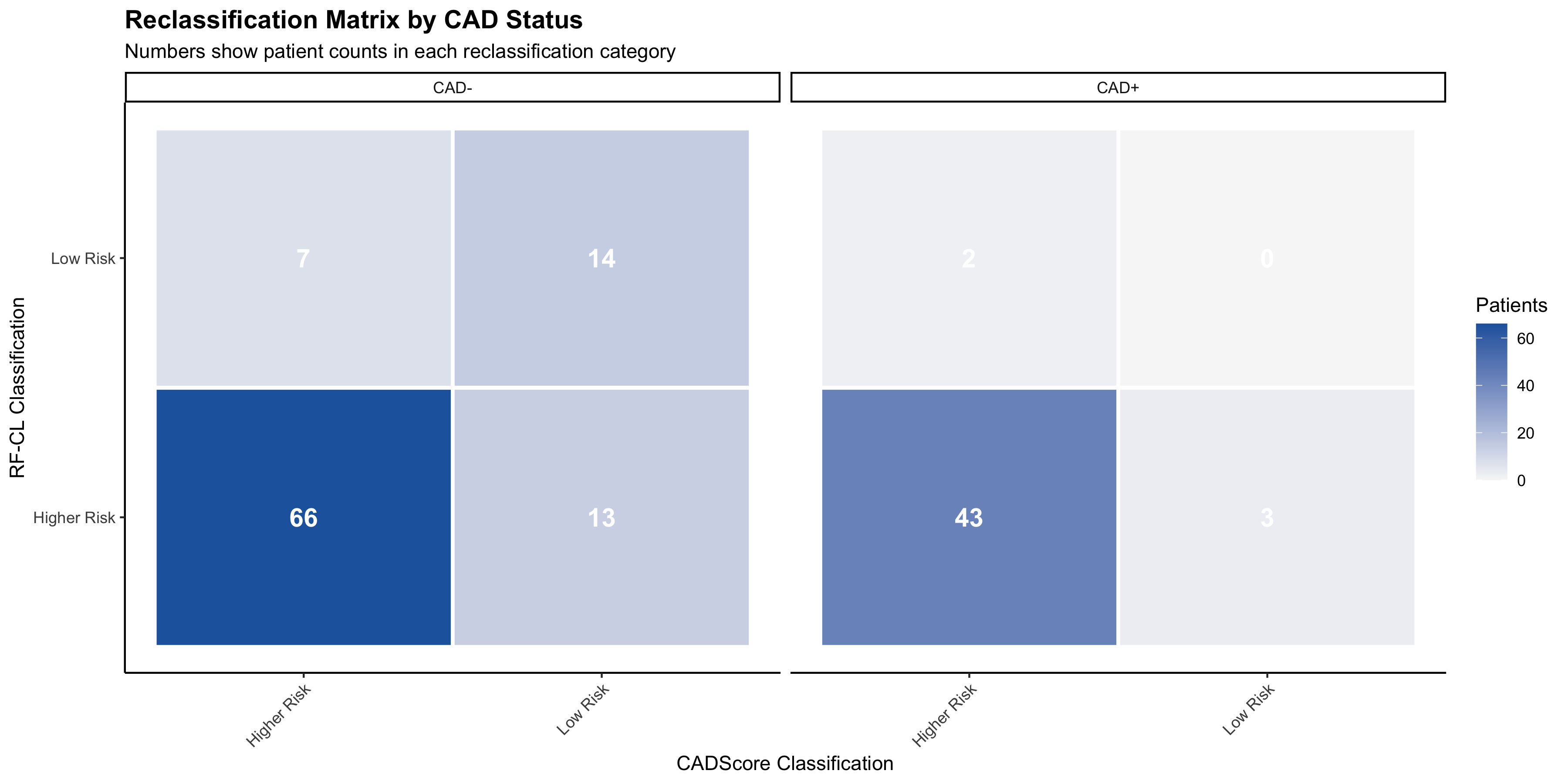


**Supplementary Figure 2:** Net Reclassification Index matrix heatmap displaying detailed cross-tabulation of patient reclassification by CAD status. The heatmap shows exact patient counts in each reclassification category, separated by cases (obstructive CAD) and controls (no obstructive CAD). Color intensity represents patient density, providing complete transparency of reclassification patterns underlying the NRI calculation.


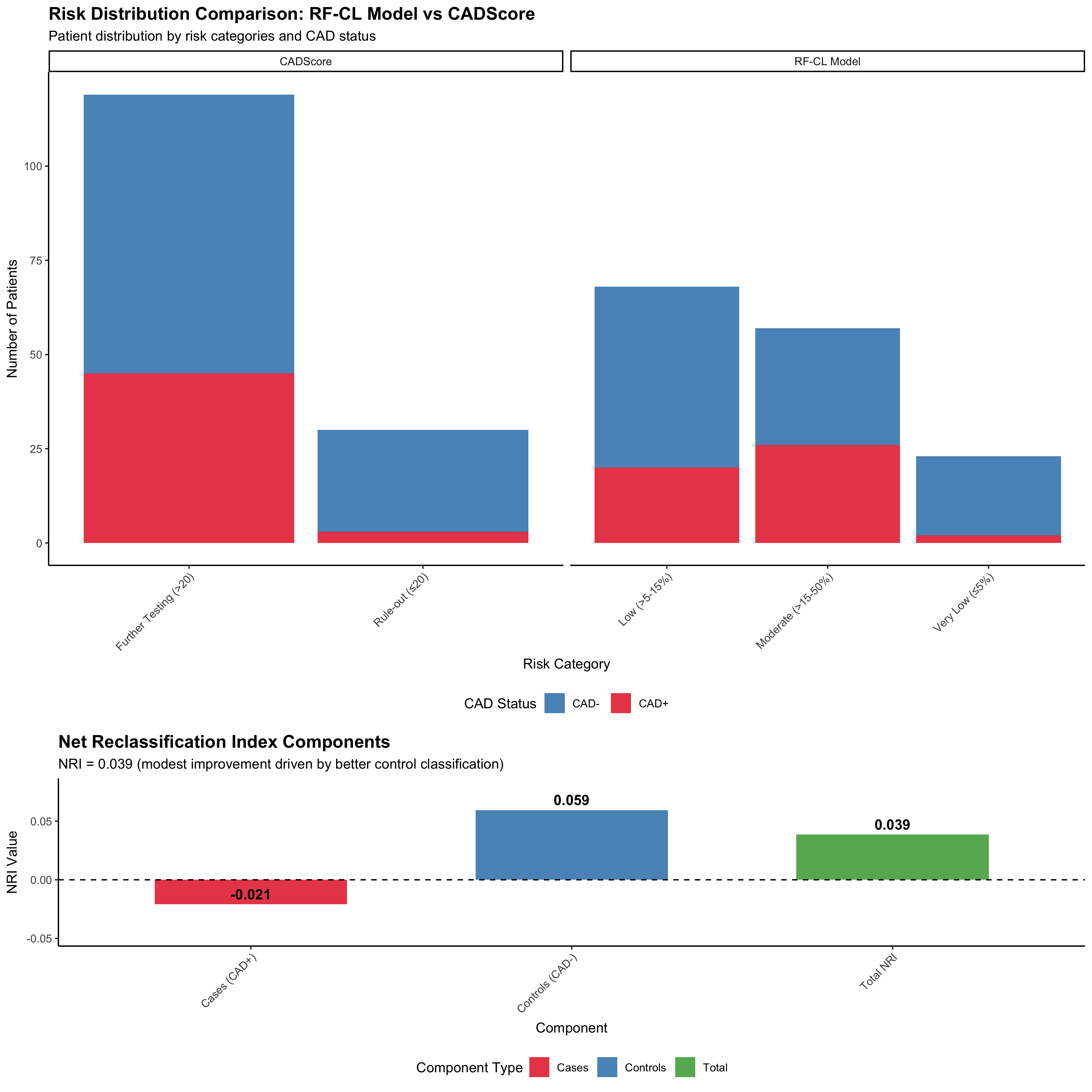


**Supplementary Figure 3:** Comprehensive Net Reclassification Index summary visualization combining risk distribution, reclassification flow, and NRI components in an integrated overview. This multi-panel figure provides a complete analytical framework for understanding the modest improvement in patient classification when using CADScore compared to the RF-CL model, supporting the quantitative NRI findings with visual evidence.


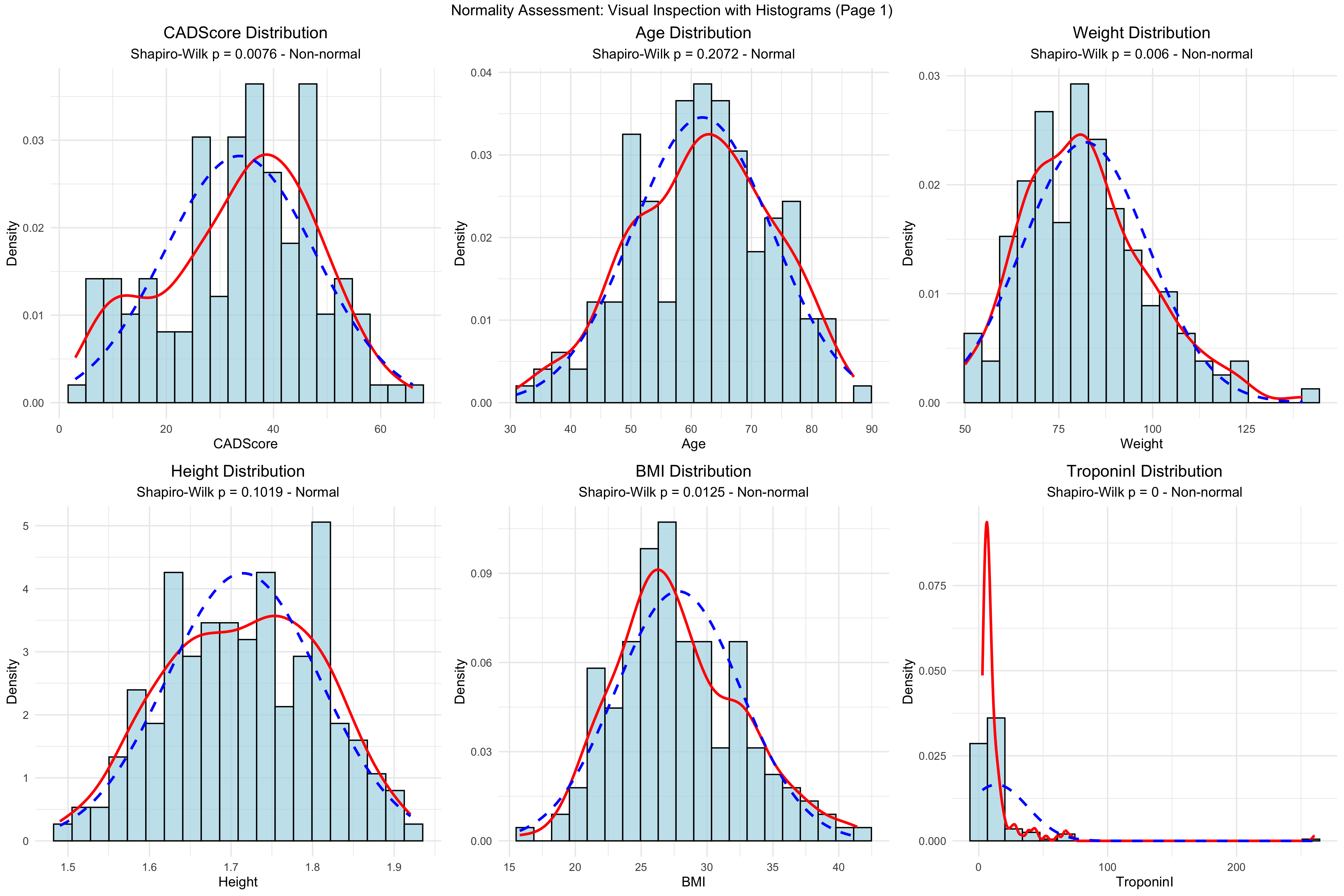


**Supplementary Figure 4:** Normality assessment histograms for key continuous variables. Visual inspection of data distributions showing histograms (light blue bars) with kernel density estimates (red lines) and normal distribution overlays (blue dashed lines). Shapiro-Wilk test results are displayed as subtitles for each variable. Variables shown include CADScore, Age, Weight, Height, BMI, and Troponin I.


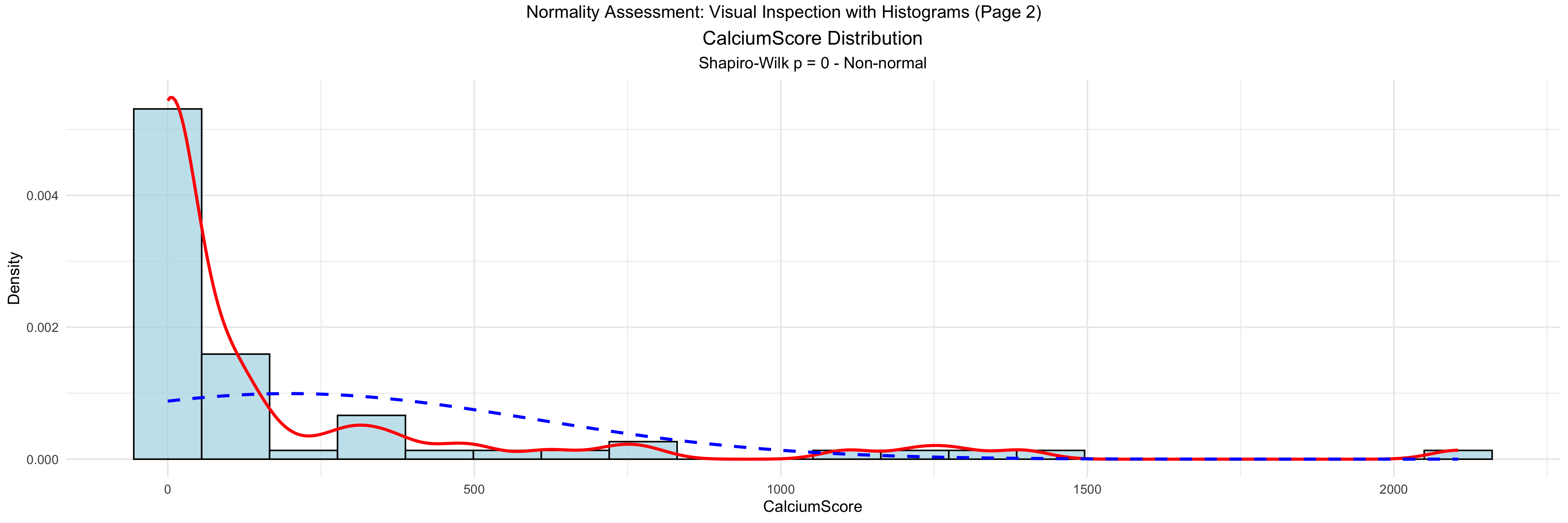


**Supplementary Figure 5:** Normality assessment histograms for key continuous variables. Continuation of visual normality assessment showing calcium score distribution.


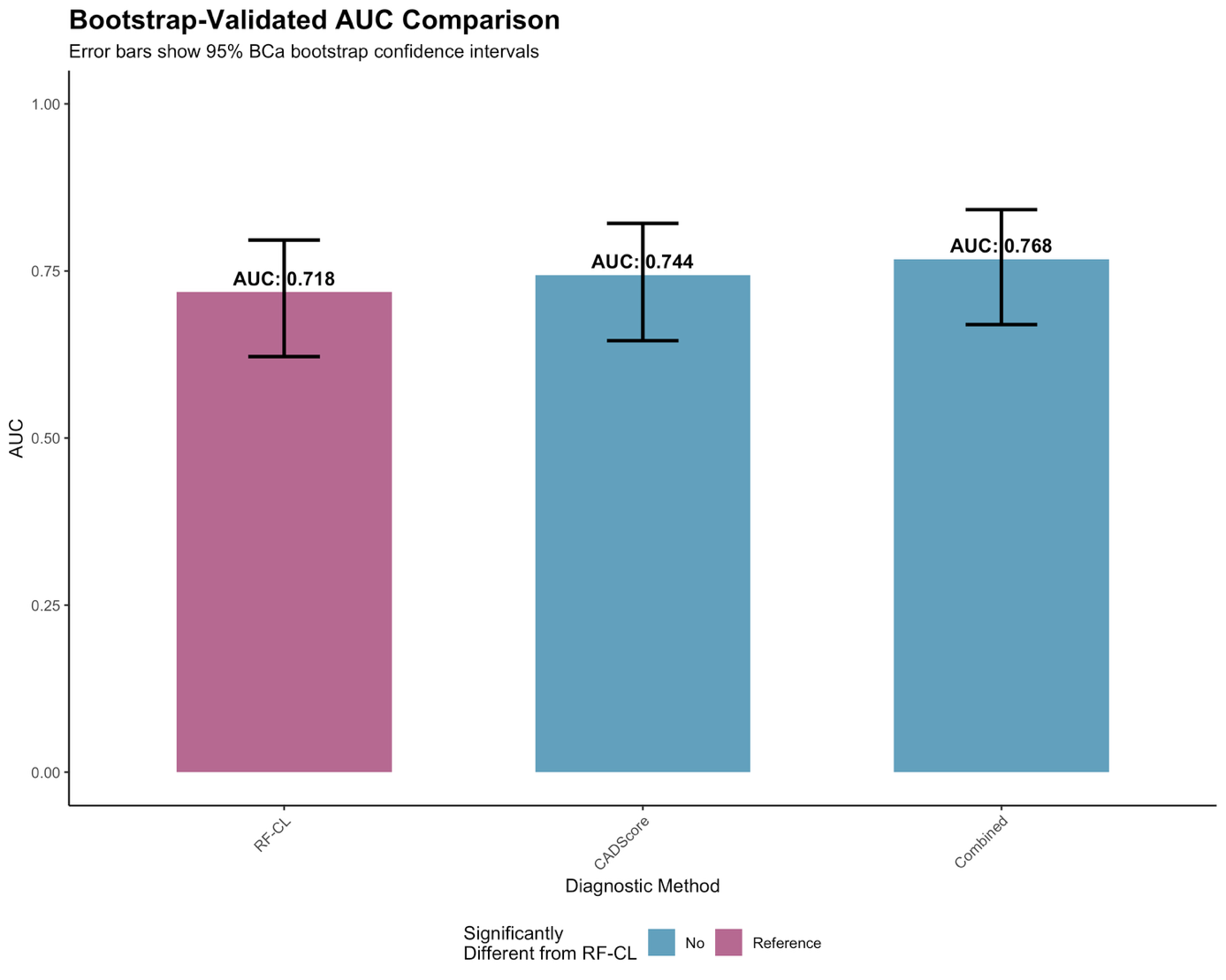


**Supplementary Figure 6:** Bootstrap-validated Area Under the Curve (AUC) comparison between diagnostic methods. Error bars represent 95% bias-corrected and accelerated (BCa) bootstrap confidence intervals based on 2000 resamples. The RF-CL model serves as the reference method (pink), while CADScore (blue) and the combined approach (blue) show overlapping confidence intervals, confirming no statistically significant differences between methods. This validation demonstrates the stability and reliability of our diagnostic performance estimates.


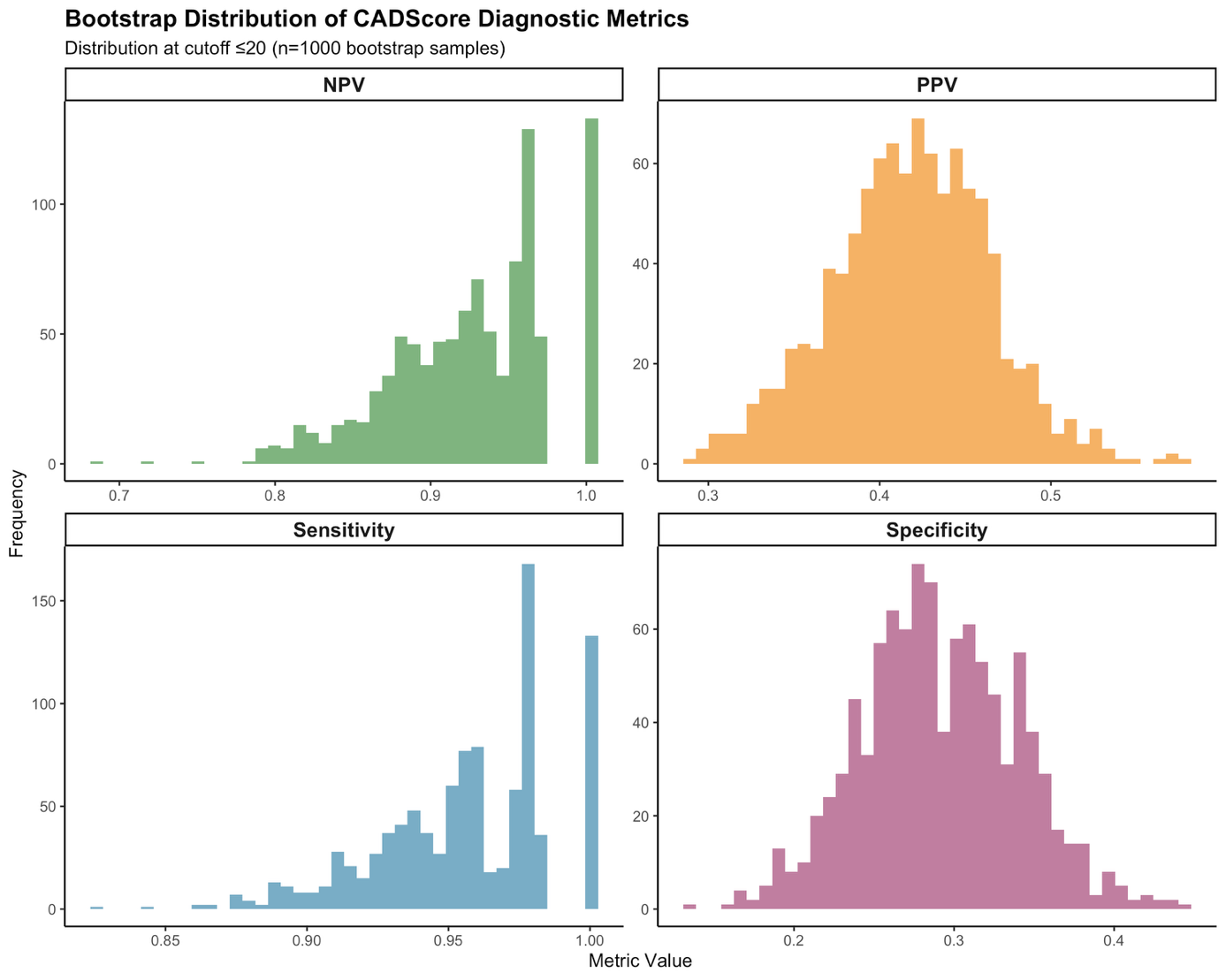


**Supplementary Figure 7:** Bootstrap distributions of key diagnostic metrics for CADScore at cutoff ≤20, based on 2000 resamples. A) Negative Predictive Value (NPV): Strongly left-skewed distribution with peak around 0.95, indicating consistent high NPV performance across bootstrap samples. B) Positive Predictive Value (PPV): Approximately normal distribution centered at 0.38-0.40, showing moderate variability. C) Sensitivity: Left-skewed distribution with peak near 0.97, demonstrating robust sensitivity performance. D) Specificity: Symmetric distribution centered at 0.28-0.30, indicating consistent but lower specificity. These distributions validate the stability of CADScore's diagnostic characteristics and provide insight into the precision of our estimates.


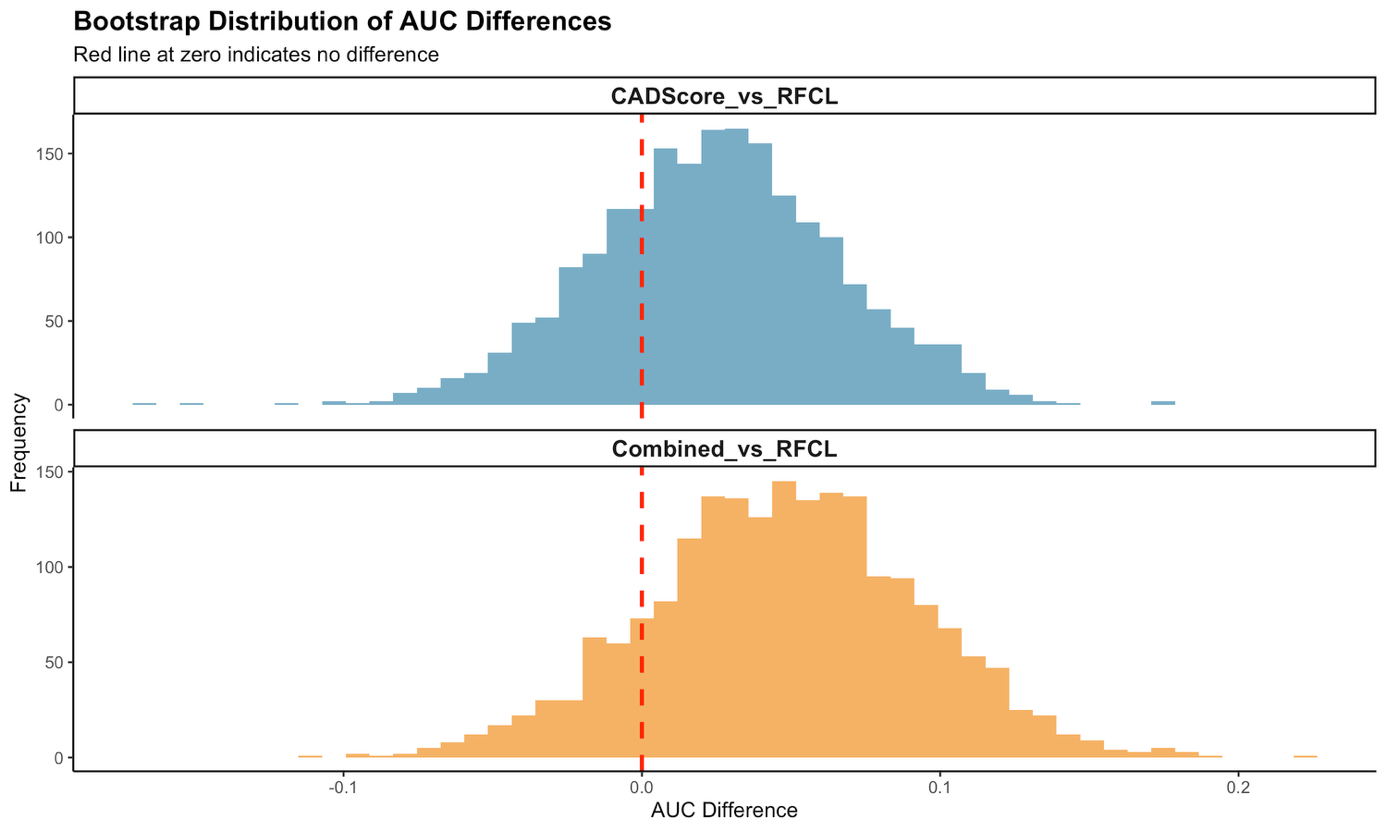


**Supplementary Figure 8:** Bootstrap distributions of AUC differences between diagnostic methods, with reference line at zero difference (red dashed line). A) CADScore vs. RF-CL: Distribution centered slightly above zero (peak around 0.02-0.05), indicating CADScore generally performs marginally better than RF-CL, though differences are small and not statistically significant. B) Combined vs. RF-CL: Broader distribution centered around 0.05-0.10, showing the combined approach provides modest improvement over RF-CL alone. Both distributions include zero within their ranges, confirming the non-significant differences observed in primary analyses. The bootstrap validation supports the robustness of our statistical conclusions.


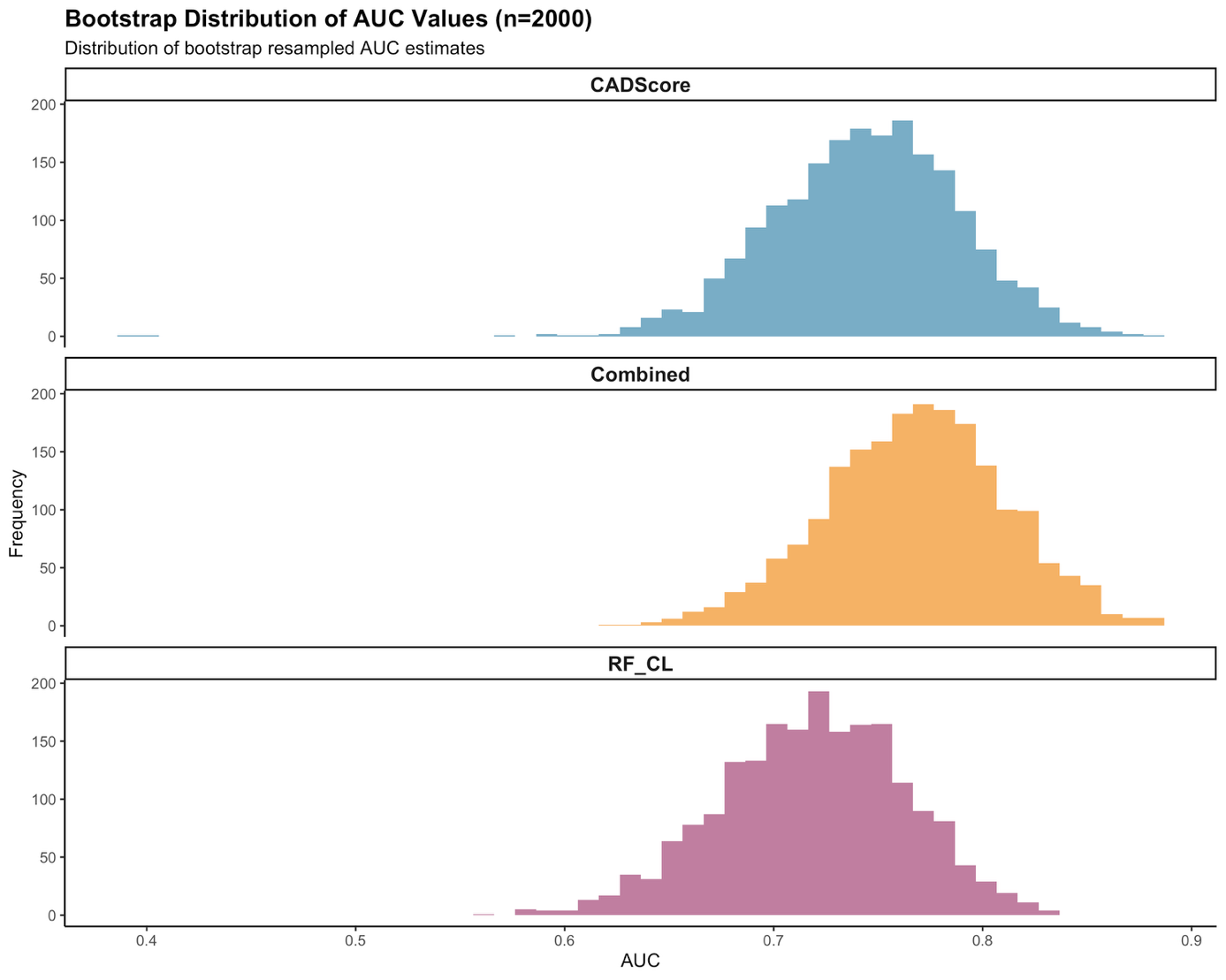


**Supplementary Figure 9:** Bootstrap distributions of absolute AUC values for all three diagnostic methods (n=2000 resamples). A) CADScore: Bell-shaped distribution with slight left skew, peak frequency around 0.70-0.75, range approximately 0.60-0.85. B) Combined approach: Narrower, more concentrated distribution with peak around 0.70-0.75, suggesting more consistent performance across bootstrap samples. C) RF-CL model: Similar distribution to combined approach, with peak slightly lower around 0.70-0.75. All distributions demonstrate good stability and reliability of AUC estimates, with overlapping ranges supporting the comparable performance between methods. The bootstrap validation confirms the robustness of our diagnostic accuracy assessments.
