# Supplementary material for: Diagnostic performance of combined biomarkers and phonocardiography vs. the 2024 ESC risk factor-weighted clinical likelihood model for detecting coronary artery disease
Source: Eur Heart J Imaging Methods Pract. 2026 Mar 10;4(1):qyag043. doi: 10.1093/ehjimp/qyag043 (PMC13032869; doi:10.1093/ehjimp/qyag043)
Supplement: qyag043_Supplementary_Data [file qyag043_supplementary_data.zip › Supplementary_Table_1.docx]

SUPPLEMENTARY TABLE 1: NET RECLASSIFICATION INDEX ANALYSIS

Supplementary Table 1: Net Reclassification Index analysis detailing patient reclassification between CADScore and RF-CL model (n=149). The table provides comprehensive cross-tabulation of patient movement between risk categories stratified by CAD status, showing exact patient counts for each reclassification pathway. Risk categories are defined as low risk (CADScore ≤20; RF-CL ≤5%) versus higher risk (CADScore >20; RF-CL >5%). NRI components are calculated separately for cases with obstructive CAD (NRI = -0.021) and controls without obstructive CAD (NRI = +0.059), yielding a total NRI of 0.039 (95% CI: -0.087 to 0.165, P = 0.548). The table includes detailed risk distribution comparison, clinical interpretation, and methodology notes supporting the quantitative NRI findings reported in the main manuscript.

## A. RECLASSIFICATION CROSS-TABLE

| **Risk Category Movement** | **RF-CL Higher Risk (>5% risk)** | **RF-CL Low Risk (≤5% risk)** | **Total** |
| --- | --- | --- | --- |
| CADScore Higher Risk (>20) | 109 | 9 | 118 |
| CADScore Low Risk (≤20) | 16 | 14 | 30 |
| Total | 125 | 23 | 148* |

*One patient had missing classification data

## B. DETAILED RECLASSIFICATION BY CAD STATUS

### CASES WITH OBSTRUCTIVE CAD (n=48)

|  | **RF-CL Higher Risk** | **RF-CL Low Risk** | **Total** |
| --- | --- | --- | --- |
| CADScore Higher Risk (>20) | 43 | 2 | 45 |
| CADScore Low Risk (≤20) | 3 | 0 | 3 |
| Total | 46 | 2 | 48 |

### CONTROLS WITHOUT OBSTRUCTIVE CAD (n=101)

|  | **RF-CL Higher Risk** | **RF-CL Low Risk** | **Total** |
| --- | --- | --- | --- |
| CADScore Higher Risk (>20) | 66 | 7 | 73 |
| CADScore Low Risk (≤20) | 13 | 14 | 27 |
| Total | 79 | 21 | 100* |

*One control had missing data

## C. NET RECLASSIFICATION INDEX COMPONENTS

**CASES (Obstructive CAD, n=48):**

- Appropriate reclassification UP (Low→Higher): 2 patients (4.2%)

- Inappropriate reclassification DOWN (Higher→Low): 3 patients (6.3%)

- Net Reclassification Index for Cases: -0.021 (-2.1%)

**CONTROLS (No Obstructive CAD, n=101):**

- Appropriate reclassification DOWN (Higher→Low): 13 patients (12.9%)

- Inappropriate reclassification UP (Low→Higher): 7 patients (6.9%)

- Net Reclassification Index for Controls: +0.059 (+5.9%)

**OVERALL NET RECLASSIFICATION INDEX:**

- Total NRI: 0.039 (3.9%)

- 95% Confidence Interval: -0.087 to 0.165

- P-value: 0.548 (non-significant)

## D. RISK DISTRIBUTION COMPARISON

**RF-CL MODEL RISK DISTRIBUTION:**

- Very Low Risk (≤5%): 23 patients (15.4%) [21 CAD-, 2 CAD+]

- Low Risk (>5-15%): 68 patients (45.6%) [48 CAD-, 20 CAD+]

- Moderate Risk (>15-50%): 57 patients (38.3%) [31 CAD-, 26 CAD+]

- High Risk (>50%): 1 patient (0.7%) [1 CAD-, 0 CAD+]

**CADSCORE RISK DISTRIBUTION:**

- Rule-out (≤20): 30 patients (20.1%) [27 CAD-, 3 CAD+]

- Further Testing (>20): 119 patients (79.9%) [74 CAD-, 45 CAD+]

## E. CLINICAL INTERPRETATION

**1. OVERALL PERFORMANCE:**

- CADScore provides modest net improvement in patient reclassification (NRI = 0.039)

- Improvement primarily driven by better classification of patients without CAD

- No statistically significant improvement (P = 0.548)

**2. RULE-OUT CAPABILITY:**

- CADScore identifies 30 patients for rule-out with 90% NPV (3/30 have CAD)

- RF-CL very low risk identifies 23 patients with 91.3% NPV (2/23 have CAD)

**3. RISK REDISTRIBUTION:**

- CADScore tends to classify fewer patients as low risk (20.1% vs 15.4%)

- More conservative approach may increase sensitivity at cost of specificity

*NRI, Net Reclassification Index; RF-CL, Risk-Factor-weighted Clinical Likelihood; CAD, coronary artery disease; NPV, negative predictive value.*
