# Supplementary material for: Diagnostic performance of combined biomarkers and phonocardiography vs. the 2024 ESC risk factor-weighted clinical likelihood model for detecting coronary artery disease
Source: Eur Heart J Imaging Methods Pract. 2026 Mar 10;4(1):qyag043. doi: 10.1093/ehjimp/qyag043 (PMC13032869; doi:10.1093/ehjimp/qyag043)
Supplement: qyag043_Supplementary_Data [file qyag043_supplementary_data.zip › Supplementary_Table_2.docx]

SUPPLEMENTARY TABLE 2: LIKELIHOOD RATIOS

Supplementary Table 2: Likelihood ratios of the CADScor©System (≤20 cutoff). PLR, Positive Likelihood Ratio, NLR, Negative Likelihood Ratio.

|  | **Value (95%CI)** |
| --- | --- |
| PLR | 1.28 (1.21–1.30) |
| NLR | 0.23 (0.04–0.93) |
