# Supplementary figures and images for: Diagnostic performance of combined biomarkers and phonocardiography vs. the 2024 ESC risk factor-weighted clinical likelihood model for detecting coronary artery disease
Source: Eur Heart J Imaging Methods Pract. 2026 Mar 10;4(1):qyag043. doi: 10.1093/ehjimp/qyag043 (PMC13032869; doi:10.1093/ehjimp/qyag043)

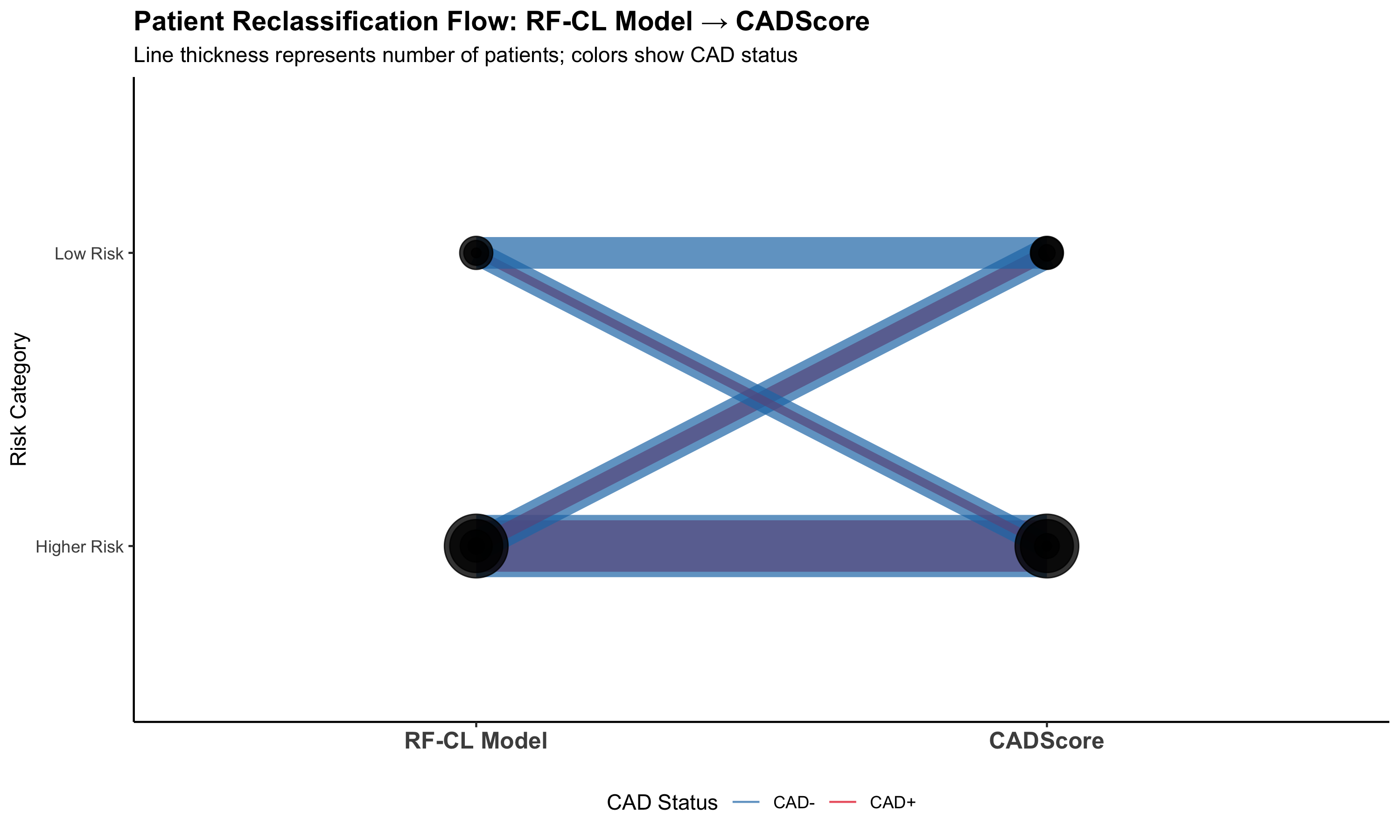

Supplement: qyag043_Supplementary_Data [file qyag043_supplementary_data.zip › Supplementary Figure 1_nri_reclassification_flow.png]

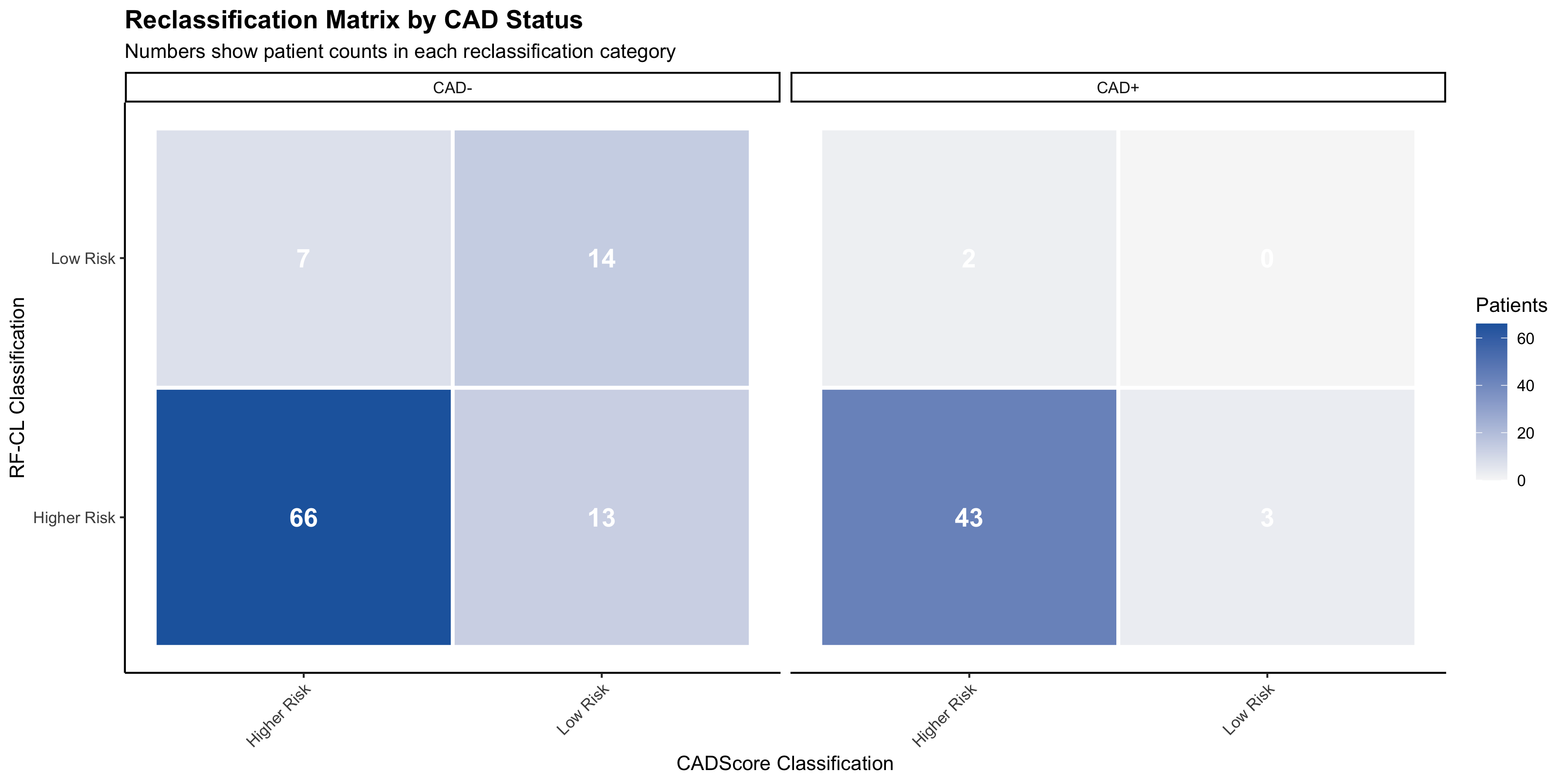

Supplement: qyag043_Supplementary_Data [file qyag043_supplementary_data.zip › Supplementary Figure 2_nri_matrix_heatmap.png]

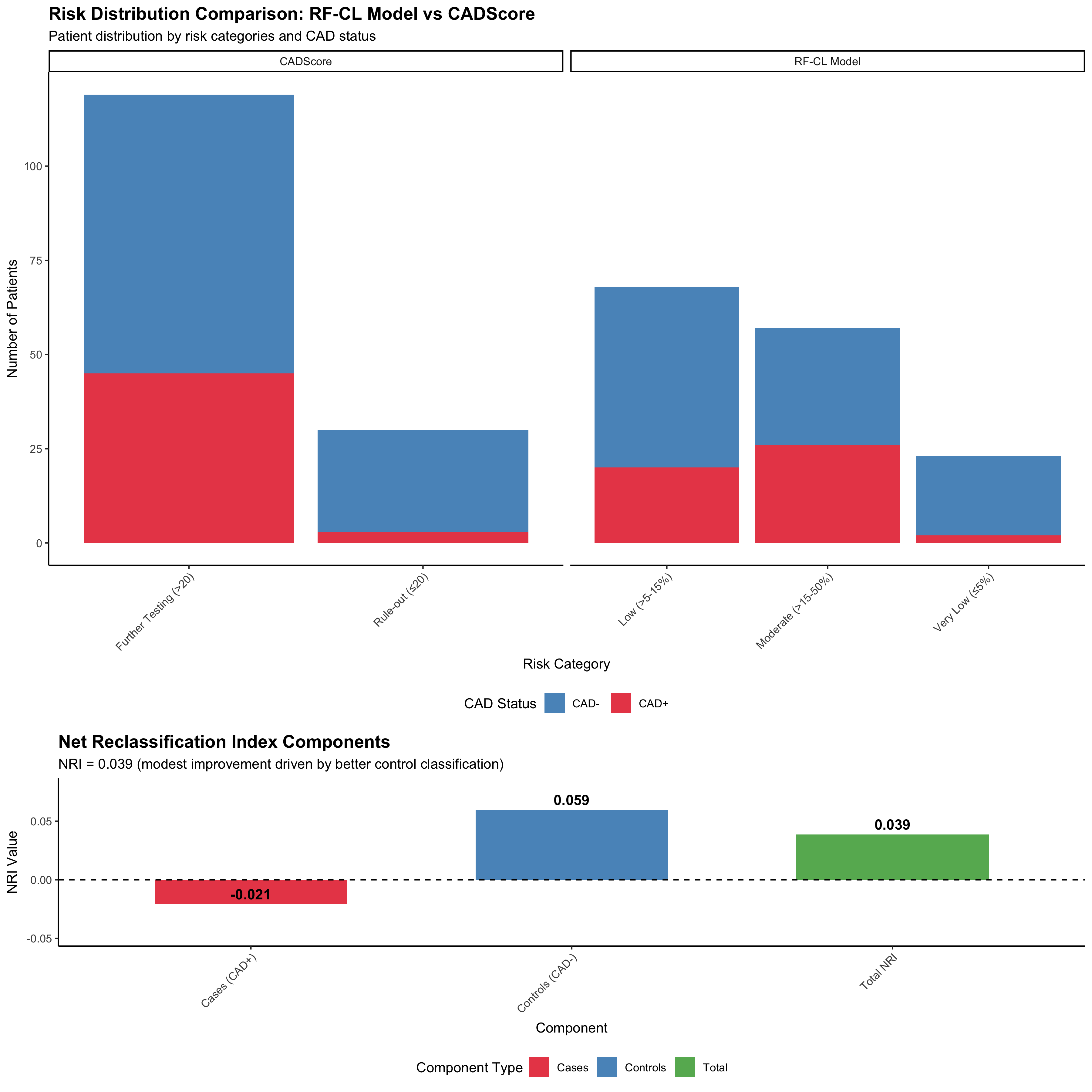

Supplement: qyag043_Supplementary_Data [file qyag043_supplementary_data.zip › Supplementary Figure 3_nri_summary_combined.png]

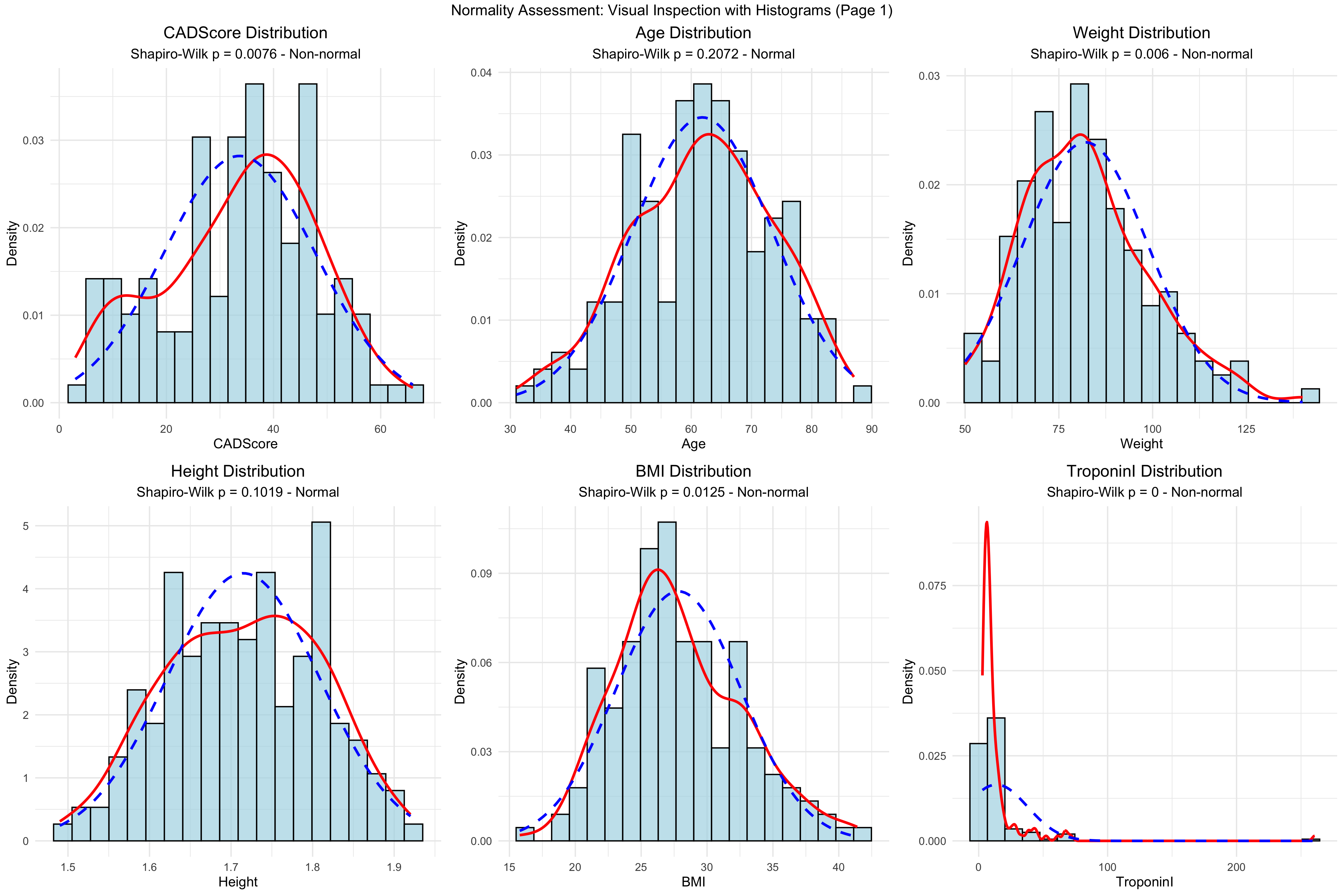

Supplement: qyag043_Supplementary_Data [file qyag043_supplementary_data.zip › Supplementary Figure 4_normality_histograms_page1.png]

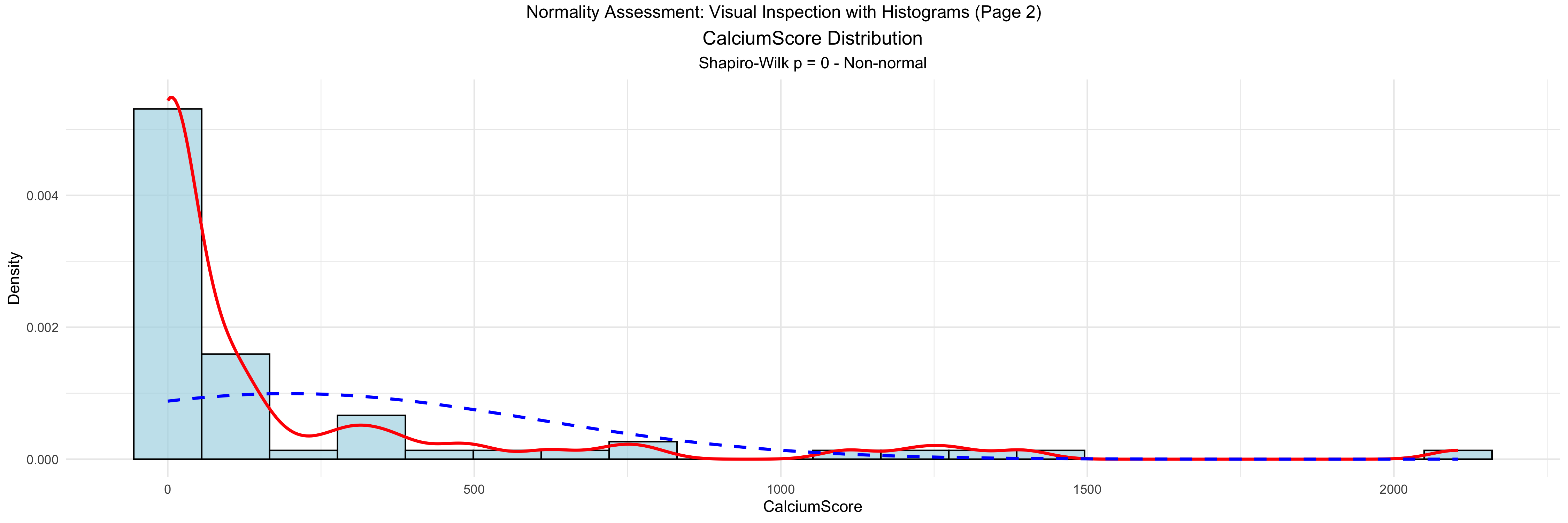

Supplement: qyag043_Supplementary_Data [file qyag043_supplementary_data.zip › Supplementary Figure 5_normality_histograms_page2.png]

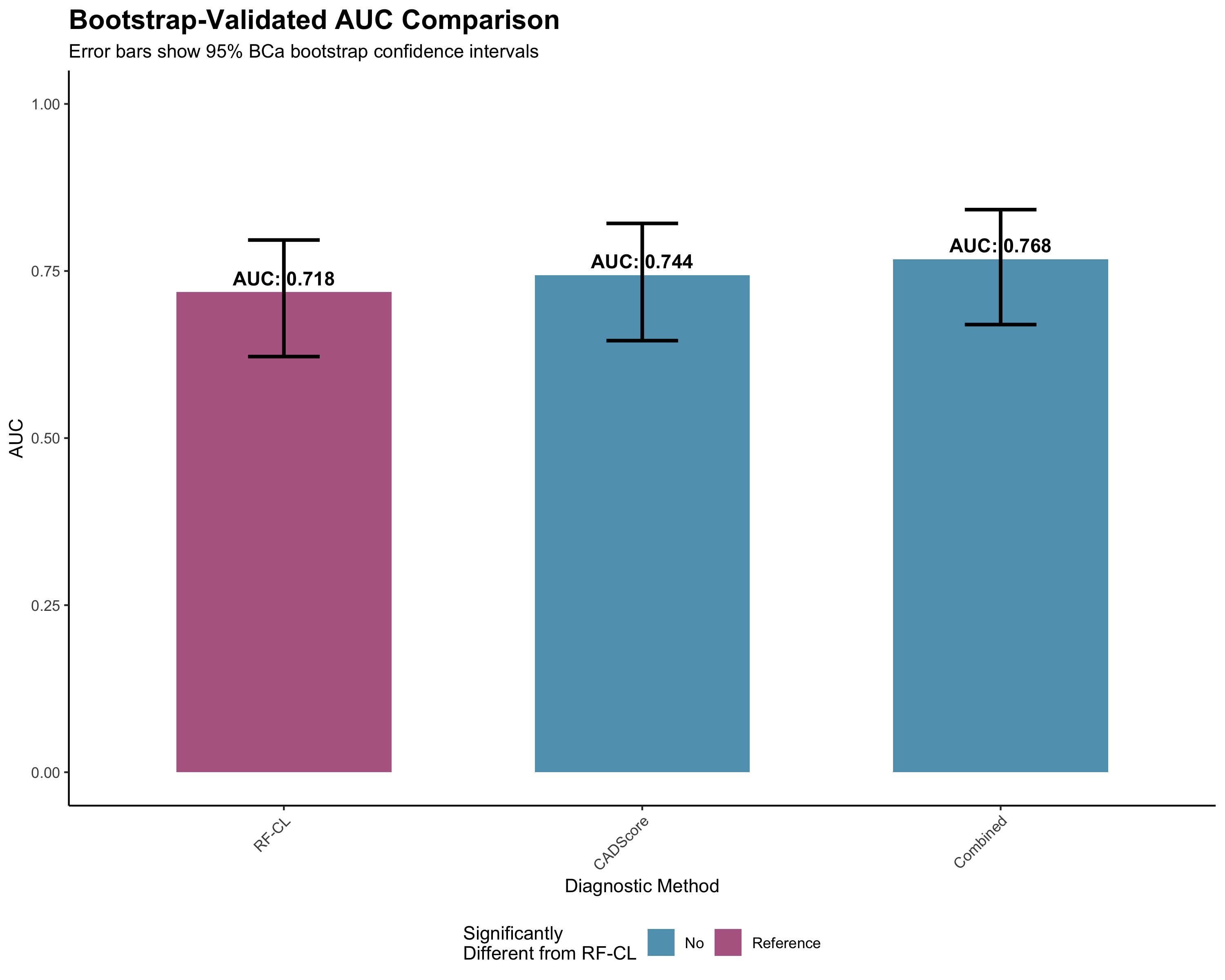

Supplement: qyag043_Supplementary_Data [file qyag043_supplementary_data.zip › Supplementary Figure 6_bootstrap_summary_comparison.png]

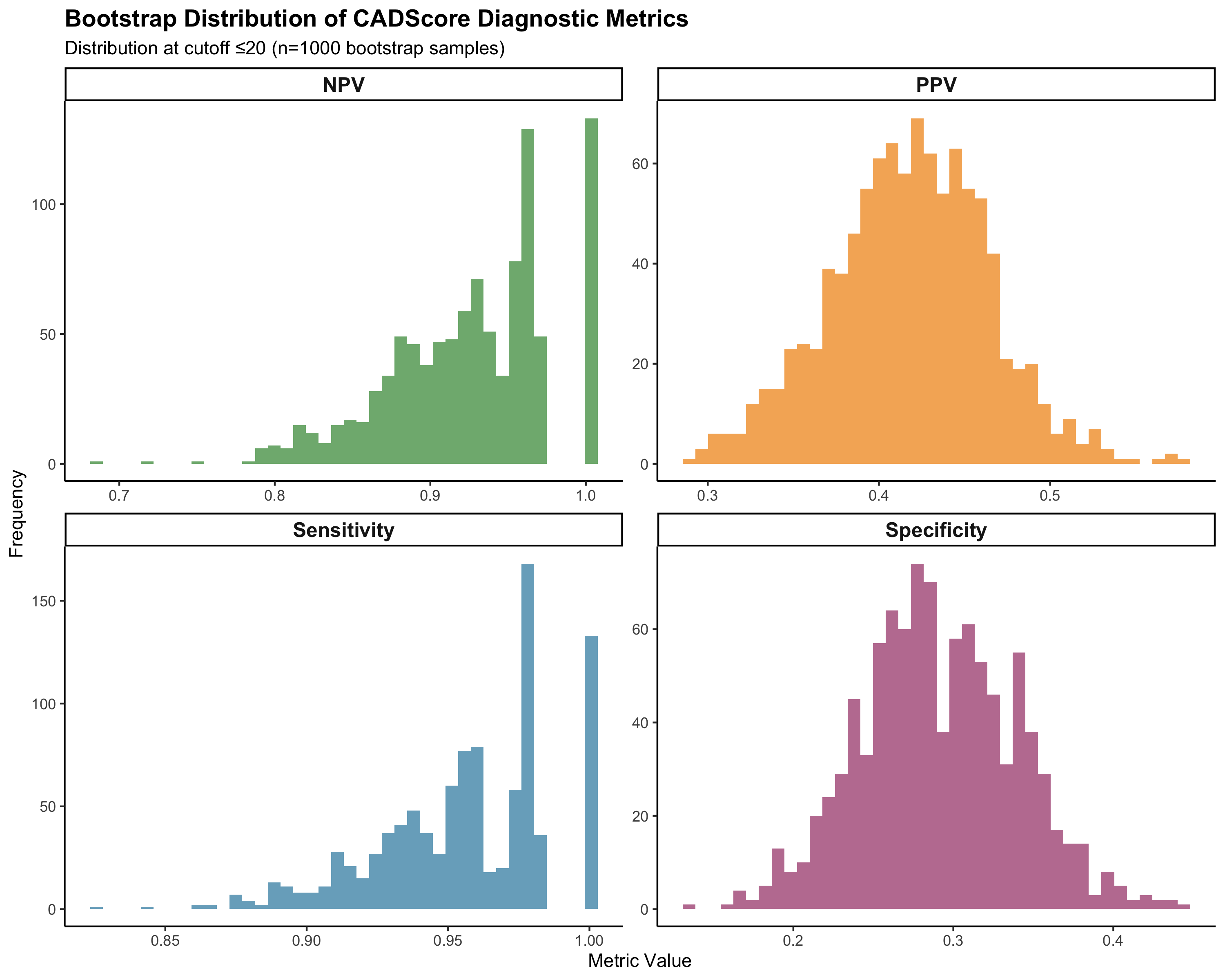

Supplement: qyag043_Supplementary_Data [file qyag043_supplementary_data.zip › Supplementary Figure 7_bootstrap_diagnostic_metrics.png]

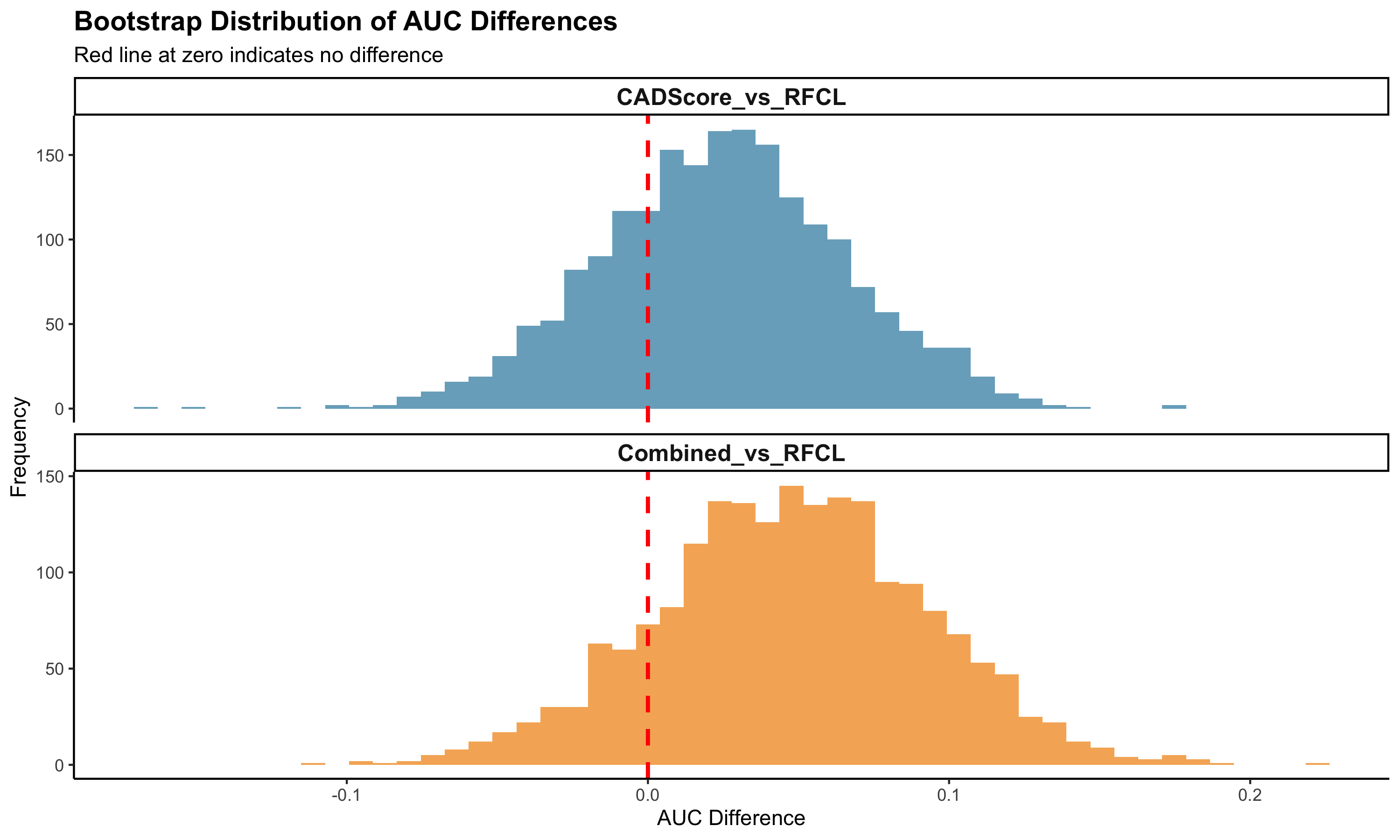

Supplement: qyag043_Supplementary_Data [file qyag043_supplementary_data.zip › Supplementary Figure 8_bootstrap_auc_differences.png]

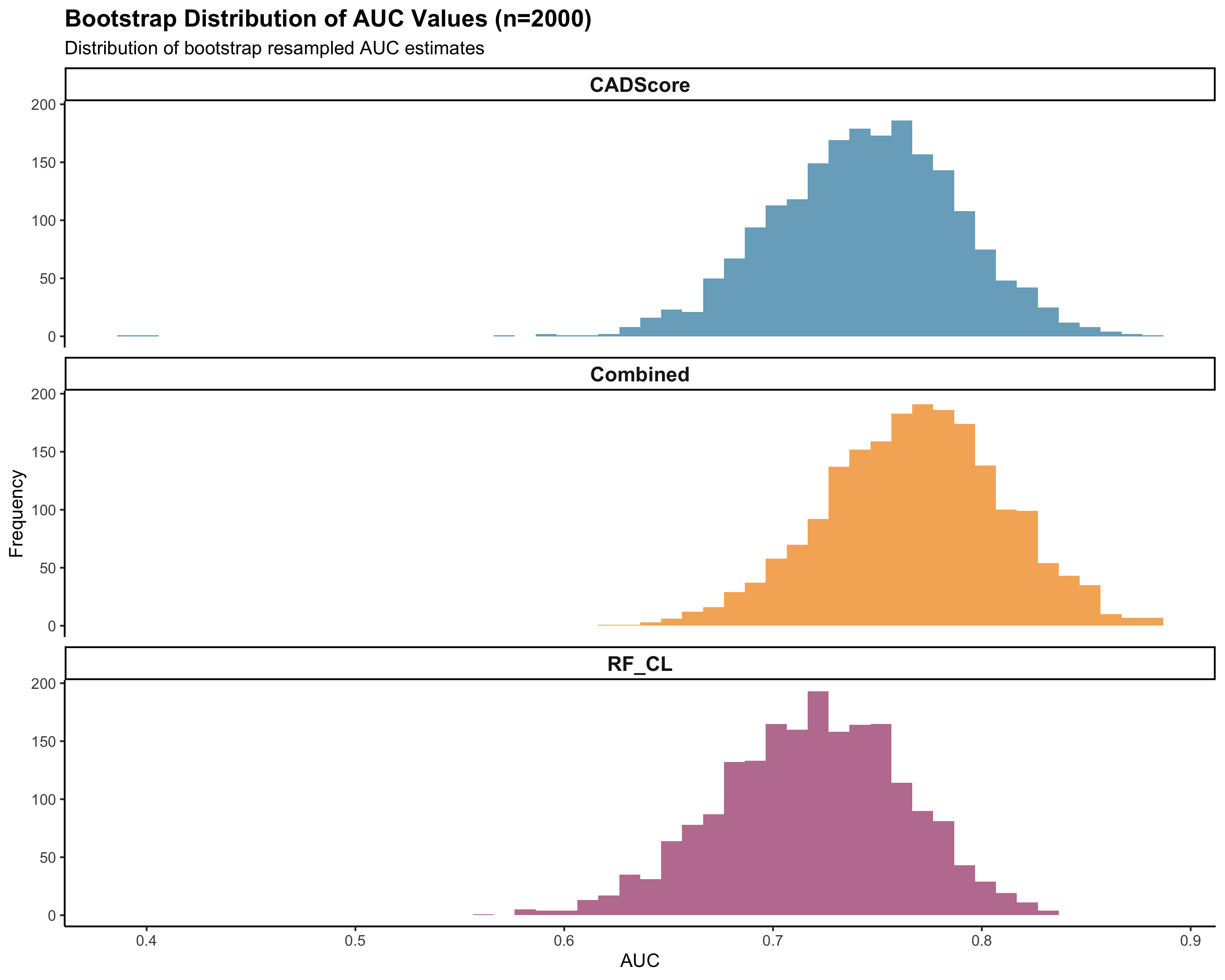

Supplement: qyag043_Supplementary_Data [file qyag043_supplementary_data.zip › Supplementary Figure 9_bootstrap_auc_distributions.png]
